# Pyrroloquinoline Quinone Is an Effective Senomorphic Agent to Target the Pro‐Inflammatory Phenotype of Senescent Cells

**DOI:** 10.1111/acel.70138

**Published:** 2025-06-19

**Authors:** Birong Jiang, Hongwei Zhang, Qixia Xu, Zhirui Jiang, Ruikun He, Qiang Fu, Yu Sun

**Affiliations:** ^1^ School of Pharmacy, Institute of Aging Medicine Binzhou Medical University Yantai Shandong China; ^2^ Center for Single‐Cell Omics, School of Public Health Shanghai Jiao Tong University School of Medicine Shanghai China; ^3^ CAS Key Laboratory of Tissue Microenvironment and Tumor, Shanghai Institute of Nutrition and Health Chinese Academy of Sciences Shanghai China; ^4^ Institute of Nutrition and Health By‐Health Co., Ltd. Guangzhou China

**Keywords:** age‐related pathologies, aging, cellular senescence, pyrroloquinoline quinone, SASP, senomorphics

## Abstract

Cellular senescence is an aging‐related mechanism characterized by cell cycle arrest, macromolecular alterations, and a senescence‐associated secretory phenotype (SASP). Recent preclinical trials established that senolytic drugs, which target survival mechanisms of senescent cells, can effectively intervene in age‐related pathologies. In contrast, senomorphic agents inhibiting SASP expression while preserving the survival of senescent cells have received relatively less attention, with potential benefits hitherto underexplored. By revisiting a previously screened natural product library, which enabled the discovery of procyanidin C1 (PCC1), we noticed pyrroloquinoline quinone (PQQ), a redox cofactor that displayed remarkable potential in serving as a senomorphic agent. In vitro data suggested that PQQ downregulated the full spectrum expression of the SASP, a capacity observed in several stromal cell lines. Proteomics data supported that PQQ directly targets the intracellular protein HSPA8, interference with which disturbs downstream signaling and expression of the SASP. PQQ restrains cancer cell malignancy conferred by senescent stromal cells in culture while reducing drug resistance when combined with chemotherapy in anticancer regimens. In preclinical trials, PQQ alleviates pathological symptoms by preventing organ degeneration in naturally aged mice while reserving senescent cells in the tissue microenvironment. Together, our study supports the feasibility of exploiting a redox‐active quinone molecule with senomorphic capacity to achieve geroprotective effects by modulating the SASP, thus providing proof‐of‐concept evidence for future exploration of natural antioxidant agents to delay aging and ameliorate age‐related conditions. Prospective efforts are warranted to determine long‐term outcomes and the potential of PQQ for the intervention of geriatric syndromes in clinical settings.

## Graphical abstract

As a natural senomorphic agent, PQQ targets the intracellular molecule. HSPA8 of senescent cells, resulting in significantly reduced expression of the SASP. PQQ holds prominent potential to delay chronological aging and ameliorate age‐related conditions by acting as an effective senomorphic agent.

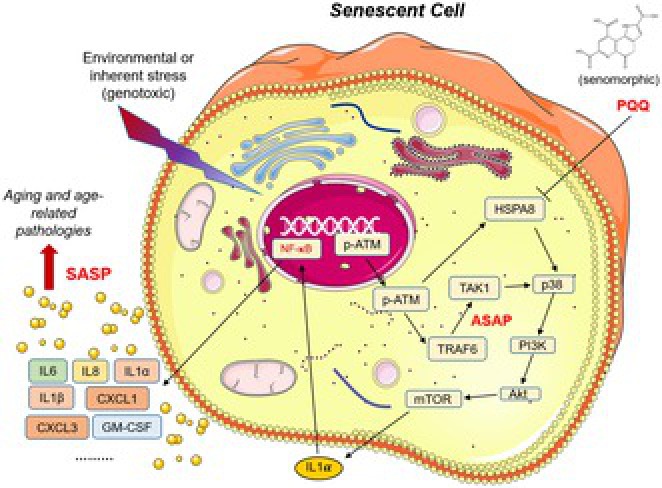

## 1 Introduction

Aging is a major risk factor for the vast majority of human chronic pathologies, including but not limited to cardiovascular diseases, neurodegenerative disorders, various cancers, osteoporosis, sarcopenia, and metabolic conditions (Costa et al. 2024; Sun et al. 2022). As one of the underlying contributors to these age‐related disorders, accumulation of senescent cells in the host microenvironment profoundly disturbs tissue homeostasis and compromises organ function (Gorgoulis et al. 2019; Huang et al. 2022). Cellular senescence is characterized by a stable cell cycle arrest, resistance to apoptosis, and, more importantly, development of a senescence‐associated secretory phenotype (SASP) (DeVito et al. 2022; Dimri and Dimri 2024). Senescent cells exhibit features associated with the G1 and G2 growth phases of the cell cycle, as well as the cell type‐ and context‐dependent SASP, hallmarks that distinguish them from both quiescent and terminally differentiated cells (Lopez‐Otin et al. 2023). Senescence is beneficial and plays a crucial role in certain situations, including embryonic development, fibrosis limitation, tissue repair, wound healing, and tumor prevention (Song et al. 2020). However, increasing lines of studies have demonstrated substantial benefits upon systemic clearance of senescent cells in both preclinical models and clinical trials, supporting the adverse influences of senescent cells, particularly those mediated by their critical hallmark, the SASP, which can generate remarkable impact on overall health conditions in a chronic manner (Farr et al. 2023).

Recent progress in understanding senescence has spurred increasing interest in the development of various approaches to target senescent cells, collectively termed senotherapy. As an emerging therapeutic modality, senotherapy represents a feasible and effective therapeutic strategy to prevent, delay, or mitigate diverse age‐related dysfunctions and disorders, thus having received intensive attention (Gasek et al. 2021). Senotherapeutic agents can either clear senescent cells or modulate SASP expression, thus minimizing the impact of the senescence‐associated pro‐inflammatory phenotype. Elimination of senescent cells is implemented by senolytics, a subclass of drugs designed to selectively target senescent cells, although the long‐term safety and efficacy of senolytics need to be clinically proven. To date, a handful of compounds have displayed senolytic properties, offering a promising solution to reverse or ameliorate age‐related conditions, as reported by multiple studies (Gomez and Jurk 2024). In contrast, naturally derived phytochemicals, including phenolic compounds and terpenes, which have antioxidant and anti‐inflammatory activities, also hold senotherapeutic potentials by targeting senescence‐regulating molecules or intracellular pathways, a subclass of agents referred to as senomorphics (Costa et al. 2024).

Technical strategies designed to screen for new senolytics involve a range of methods to induce cellular senescence, select target cell types, and evaluate the senolytic efficacy. The choice of methods may substantially influence the outcomes of screening, although high‐quality screens are usually featured with robust systems, adequate controls, and systematic validations by multiple assays (Suda et al. 2024). In contrast, the development of senomorphics is tactically more straightforward and technically less challenging, as the evaluation criteria basically rely on the overall effect of candidate agents in controlling the expression or formation of a full‐spectrum SASP (Costa et al. 2024; Liu et al. 2024). In terms of the functional role, senomorphics mainly modulate the secretory activity of senescent cells without directly and radically eliminating senescent cells per se. These agents specifically target intracellular pathways, such as those implicating ATM, TAK1, p38MAPK, mTOR, JAK/STAT, and the NF‐κB complex (Sun et al. 2022). Some FDA‐approved clinical agents such as metformin and rapamycin, exhibit robust senomorphic effects (Laberge et al. 2015; Moiseeva et al. 2013). Although senomorphics generally require continuous rather than intermittent administration, these agents hold the advantage of reducing inflammation, minimizing tissue damage, and controlling senescence‐associated adverse effects while causing fewer off‐target effects, particularly systemic cytotoxicity, when administered in vivo (Chaib et al. 2022).

PQQ (4,5‐dihydro‐4,5‐dioxo‐1H‐pyrrolo [2,3‐f]quinoline‐2,7,9‐tricarboxylic acid) is an aromatic and water‐soluble quinone with chemical properties analogous to that of the combination of ascorbic acid, riboflavin, and pyridoxal‐5‐phosphate (Jonscher et al. 2021). Being highly electrophilic and holding the capacity to react with diverse substances, PQQ can serve as a dehydrogenase cofactor, a free radical scavenger, and an amine oxidase catalyst, while both its oxidized (PQQ_ox_) and reduced (PQQ_H2_) forms are involved in the redox cycling of eukaryotic cells (Takeda et al. 2019). PQQ attenuates multiple pathophysiological dysfunctions, including those associated with inflammation, ischemia, and lipotoxicity (Gao et al. 2022a). By acting as an oxidoreductase cofactor, PQQ exhibits a protective role in premature senescent HEI‐OC1 auditory cells, whereby it activates the SIRT1/PGC‐1α signaling pathway, improves mitochondrial structure, and enhances mitochondrial respiratory capacity (Gao et al. 2022a). A recent study reported that PQQ inhibits aging‐associated intervertebral disk degeneration (IVDD) by reducing oxidative stress, attenuating cellular senescence, and matrix degradation via Nrf2 activation, supporting PQQ as a potential agent for clinical prevention and treatment of natural aging‐related IVDD (Xue et al. 2024). However, the effects of PQQ on senescent cells, particularly their survival, secretory activity, and, more importantly, the molecular targets that mediate these effects, remain hitherto underexplored. In this study, we examined the influence of PQQ on senescent human stromal cells, determined the optimal concentration of PQQ, and investigated its overall impact on cellular senescence, specifically the SASP development. We found that PQQ effectively dampens the pro‐inflammatory phenotype of senescent cells, minimizes the impact of these cells on the surrounding microenvironment, improves chemotherapeutic efficacy, and ameliorates age‐related structural disorder of several major organs. The discovery of PQQ's geroprotective properties may make it an exploitable therapy for alleviating age‐related diseases, providing a rapid and cost‐saving opportunity for clinical translation.

## 2 Results

### 2.1 Drug Screening Identifies PQQ as a Potential Senomorphic Agent

**FIGURE 1:**
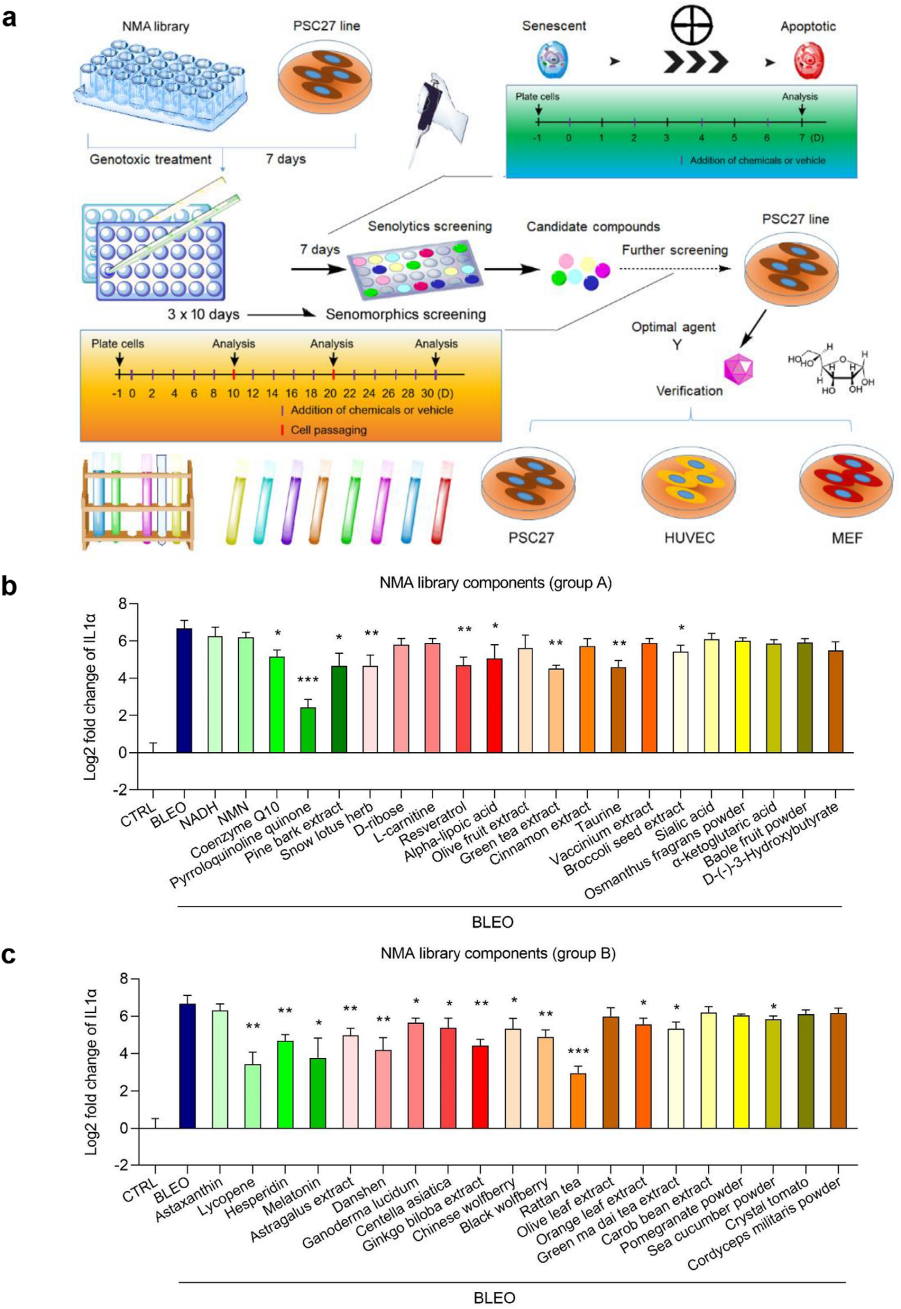
In vitro screening for senotherapeutic candidates with a natural medicinal agent (NMA) library. (a) A schematic diagram of a stromal cell‐based screening strategy for an NMA library composed mainly of 46 naturally derived agents. Upon completion of the 1st round of senolytics screening, all agents were subjected to the 2nd round of senomorphics screening. The senotherapeutic potential of candidate agents was then validated in several stromal cell lines. (b) Assessment of the effects of individual NMA agents (group A, 21) on the expression of IL‐1α, a core SASP factor, in CTRL, SEN, and SEN cells treated with various agents in culture. (c) Evaluation of the effects of individual NMA agents (group B, 20) on the expression of IL‐1α in CTRL, SEN, and SEN cells treated with various agents in culture. In (b) and (c), each agent was applied at 1 μg/mL. CTRL, control. SEN, senescent. Data in (b) and (c) are shown as mean ± SD and representative of three independent biological replicates, with *p* values calculated by Student's two‐sided *t*‐tests. *p < 0.05; **p < 0.01; ***p < 0.001.

To discover new compounds that can be used to specifically and effectively target senescent cells, we conducted an unbiased drug screening with a library composed of 46 natural medicinal agents (NMAs), most of which are phytochemical products (Table S1). For this purpose, we selected a primary normal human prostate stromal cell line, namely PSC27, as a cell‐based model. PSC27 consists primarily of fibroblasts but contains a minor percentage of non‐fibroblast cell lineages such as endothelial cells and smooth muscle cells (Sun et al. 2012). Upon exposure to environmental stressors such as genotoxic chemotherapy, H_2_O_2_, or ionizing radiation, PSC27 develops a typical SASP, making it an ideal and robust model for studying senescence and exploring geroprotective interventions (Chen, Long, et al. 2018; Zhang et al. 2018). To induce cellular senescence, we treated PSC27 with a pre‐optimized sublethal dose of bleomycin (BLEO; 50 μg/mL) and observed increased staining positivity of senescence‐associated β‐galactosidase (SA‐β‐gal), decreased BrdU incorporation, and enhanced DNA damage response (DDR) foci 7–10 days afterwards (Liu et al. 2024). To streamline the technical procedure, we developed a screening strategy to appraise the effect of individual natural agents generated on the in vitro survival and expression profile of senescent PSC27 cells (Figure 1a; Geng et al. 2019; Liu et al. 2024).

Upon exploration of the potential of PSC27 as an experimental cell model for scaled‐up drug screening, we determined the efficacy of these individual NMA components in serving as senolytics, or alternatively, their potential in selectively killing senescent cells. Natural agents in the NMA library displaying senolytic activity were generally excluded from further investigations, including grape seed extract, quercetin, and curcumin, which have been studied intensively by multiple research groups in recent years (Figure S1a,b). In this study, we chose to focus on exploring the potential of the remaining NMA agents in serving as senomorphics, or more specifically, in inhibiting SASP expression. We chose to use IL‐1α, a typical hallmark SASP factor, as the detection target or evaluation index for transcription‐based quantitative assays. Of note, there were quite a few NMA components that displayed the efficacy of suppressing the expression of IL‐1α in a statistically significant manner (Figure 1b,c). Among these agents, PQQ exhibited a prominent potential in downregulating IL‐1α expression, with the fold change or downregulation extent generally superior to other agents (Figure 1b,c).

Of note, PQQ possesses a special molecular structure that is distinct from other established cofactors like vitamins and minerals (Figure S2a–c). With its unique pyrroloquinoline ring system, PQQ serves as a cofactor for several redox‐activated enzymes and plays a crucial role in various physiological processes (Yan et al. 2024), while its potential in targeting senescent cells is largely unknown.

### 2.2 PQQ Downregulates Expression of the SASP Without Interfering With Cellular Senescence

**FIGURE 2:**
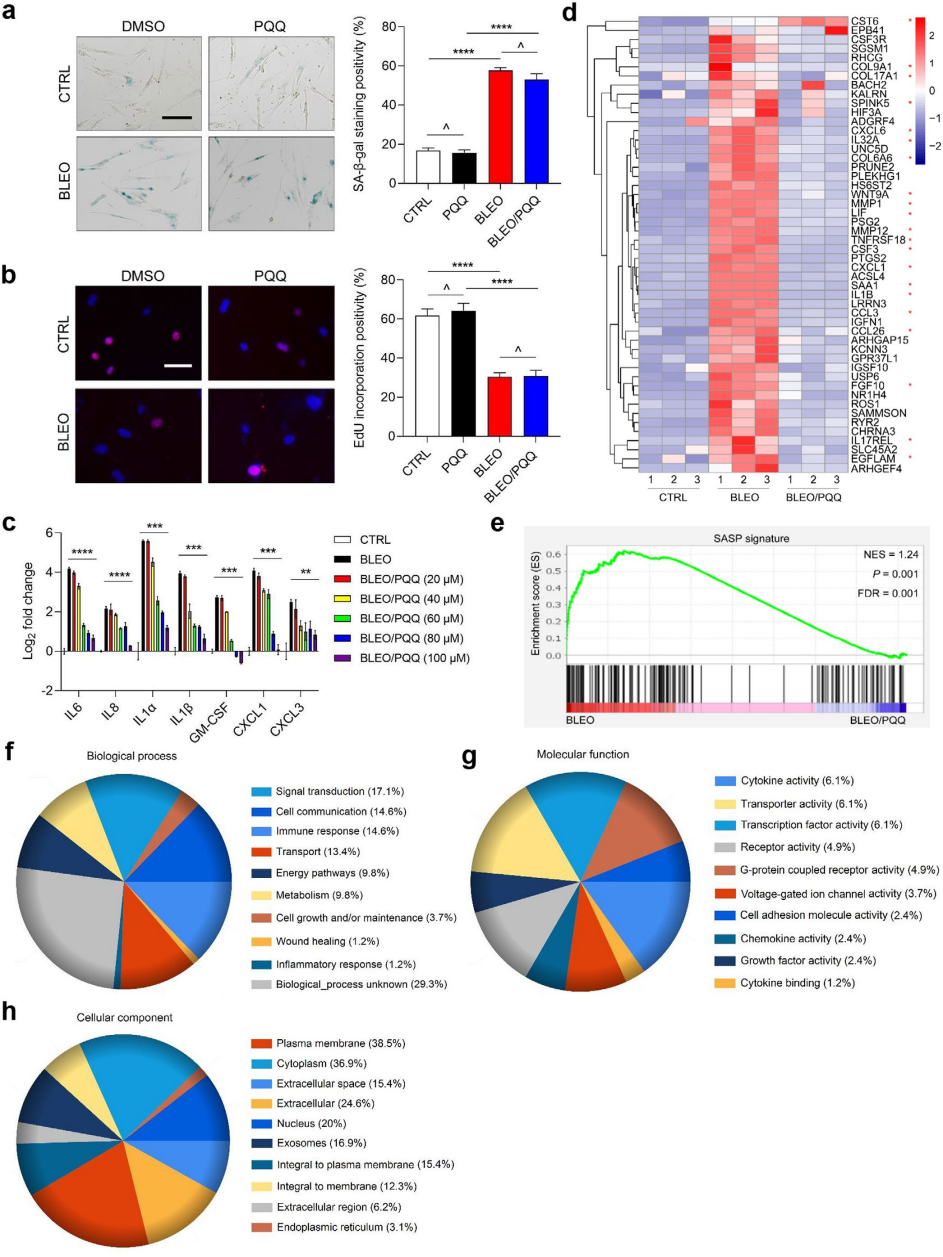
Examination of PQQ effects on cellular senescence and expression profile of senescent cells. (a) Cellular senescence determination by SA‐β‐gal staining. Left, representative images of SA‐β‐gal staining. Right, comparative statistics. Scale bar, 20 μm. (b) Cell cycle arrest evaluation by EdU staining. Left, representative images of EdU staining. Right, comparative statistics. Scale bar, 15 μm. (c) Quantitative transcriptional analysis of core SASP factor expression upon BLEO‐induced senescence in the absence or presence of PQQ applied at increasing concentrations. (d) Heatmap displaying the expression landscape of human stromal cells (PSC27) upon senescence and/or PQQ intervention. Human genes were ordered and clustered according to fold change of upregulation in CTRL versus SEN cells and their functional relevance, with corresponding changes upon PQQ treatment exhibited side‐by‐side. PQQ was applied at a concentration of 100 μM. (e) GSEA output presentation of the enrichment of a significant gene set indicative of the development of a typical SASP. (f–h) Pie charts displaying the biological process (f), molecular function (g) of cellular component (h) of top 100 genes that were most downregulated upon treatment by PQQ in senescent PSC27 cells. For all datasets, cells were collected for analyses 7–8 days after senescence induction by BLEO or 3 days after PQQ treatment of senescent cells in culture condition. Data in (a–c) are shown as mean ± SD and representative of three independent biological replicates. *p* values calculated by two‐sided *t*‐tests. ^*p* > 0.05; *p < 0.05; **p < 0.01; ***p < 0.001; ****p < 0.0001.

To evaluate the overall effect of PQQ on senescent cells, we first performed assays to determine its potential impact on cellular senescence. To this end, we conducted in vitro assays by assessing cellular senescence and analyzing cell cycle progression. The results showed that neither SA‐β‐gal staining nor EdU incorporation was significantly altered by PQQ, no matter whether cells were proliferating or senescent. For instance, in the case of BLEO‐induced senescence (Figure 2a,b).

Among the concentrations of PQQ we examined, 100 μM of PQQ caused the most remarkable decline in the expression of a subset of core SASP factors, including IL‐6, IL‐8, IL‐1α, IL‐1β, GM‐CSF, CXCL1, and CXCL3 (Figure 2c). While higher concentrations may further restrain the SASP, proliferating cells can also be affected. RNA‐seq data indicated that PQQ at 100 μM effectively downregulated expression of the vast majority of SASP factors, although some genes not directly linked to senescence or the SASP also displayed a certain extent of changes (Figure 2d). Furthermore, results from gene set enrichment analysis (GSEA) supported the role of PQQ in modulating the hallmark signature of the SASP (Figure 2e).

Upon further assessment by mapping transcripts to a gene ontology (GO) database comprising HPRD, Entrez Gene, and UniProt accession identifiers (Maglott et al. 2011; UniProt 2010), we noticed that the most prevalent biological processes correlated with upregulated genes (top 100 selected as representatives) were signal transduction, cell communication, and immune response (Figure 2f). The most predominant molecular functions of bioactive proteins encoded by these genes encompassed cytokine activity, transport activity, transcription factor activity, and G‐protein‐coupled receptor activity (Figure 2g). Additionally, the most typical cellular components related to the upregulated genes involved the plasma membrane, cytoplasm, and extracellular space (Figure 2h). Together, these data suggest a prominent capacity of PQQ in restraining the expression of genes inherently correlated with pro‐inflammatory phenotype and secretory activity of senescent cells, with the biochemical nature and intracellular activity of most of these genes falling in the range of a full‐spectrum SASP.

Alternatively, to enhance the reproducibility of our findings, we induced cellular senescence through multiple strategies, including replicative senescence (RS) and oncogenic *HRAS* ^*G12V*^‐induced senescence (OIS). Across these individual conditions, PQQ consistently downregulated the expression of typical SASP factors in senescent cells (Figure S3a,b). To further validate these results, we replicated the assays using human umbilical vein endothelial cells (HUVECs) and mouse embryonic fibroblasts (MEFs), which display hallmark features of cellular senescence such as growth arrest, reduced proliferation, and SASP factor secretion upon environmental or inherent stress (Huang et al. 2024; Sax et al. 2024). The data obtained from both HUVECs and MEFs closely mirrored those acquired from PSC27 cells, confirming that PQQ broadly inhibits SASP expression, regardless of senescence induction modality and cell type examined (Figure S3c–h).

### 2.3 PQQ Targets the Intracellular Molecule HSPA8 and Interferes With SASP Development

**FIGURE 3:**
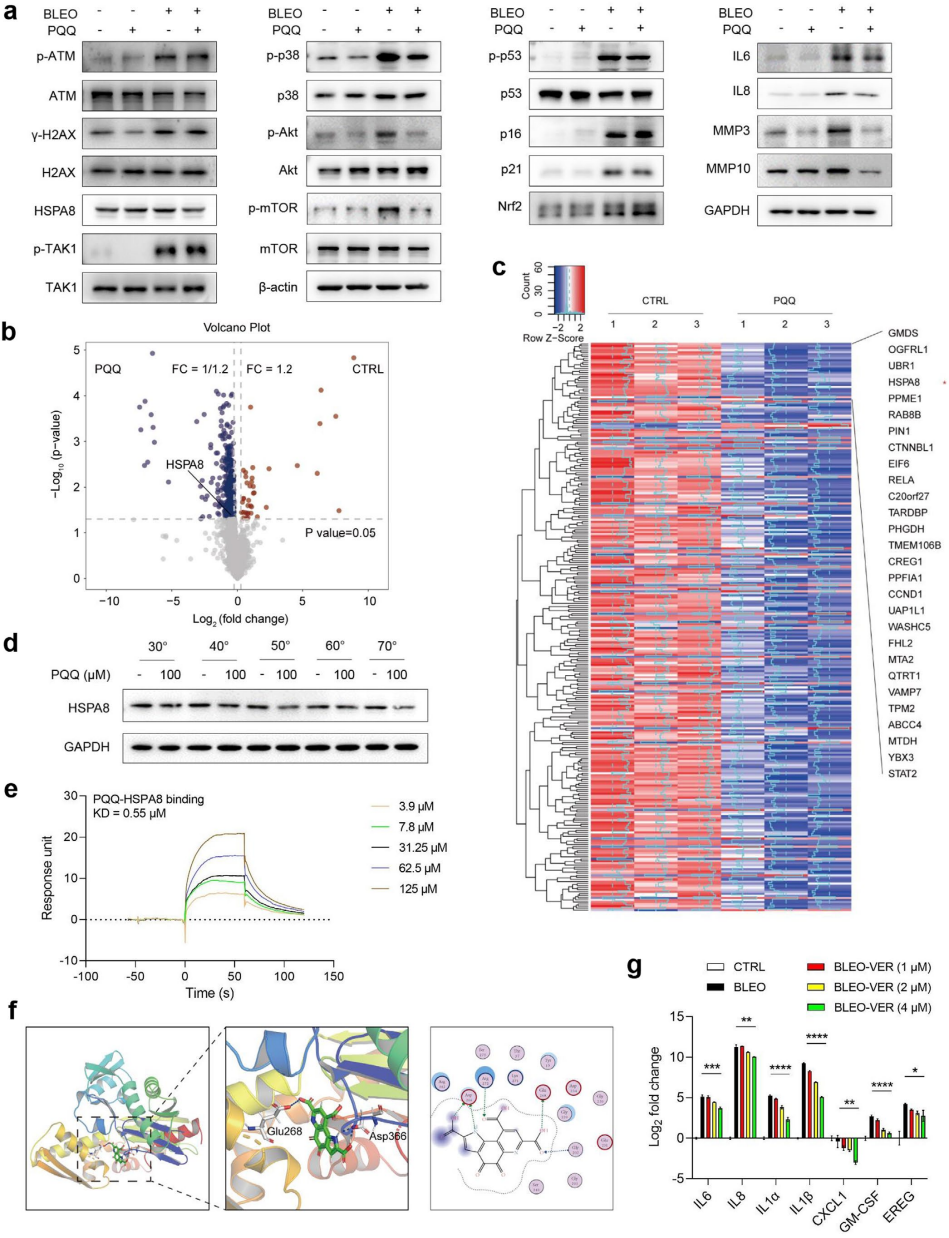
Characterization of the molecular mechanism supporting PQQ to curtail SASP expression in senescent cells. (a) Immunoblot assay of several key factors of functional relevance to regulate the SASP development. Senescence of PSC27 cells was induced by treatment with BLEO. GAPDH, loading control. (b) Volcano plot displaying the significantly differentially regulated proteins (Red, upregulated; Blue, downregulated) in the DARTS assay. Senescent stromal cell lysis solution was treated with DMSO (CTRL) or PQQ. (c) A clustering heatmap that profiles proteomic expression change as depicted in (b). Red stars denote PQQ target proteins that may play essential roles in mediating its effect on senescent cells. (d) CETSA assay evaluation of the thermal stabilization of HSPA8 upon incubation with PQQ at a gradient range of temperatures from 30°C to 70°C in protein lysates of PSC27 cells. (e) SPR demonstration that HSPA8 directly binds to PQQ. In the SPR assay, HSPA8 was treated with PQQ over a range of concentrations from 3.25 to 125 μM. KD value is shown beside the traces. SPR, surface plasmon resonance. (f) In silico molecular modeling of PQQ bound to Glu268 and Asp366 of HSPA8. Reported X‐ray diffraction microscopy structure (PDB ID: 6ZYJ) was applied to perform molecular docking, with Glu268 and Asp366 highlighted in the structure. The structure of PQQ is provided at the right, with residues that potentially interact with HSPA8 illustrated. Docking between HSPA8 and PQQ: *S* = −6.360 kcal/mol. (g) Quantitative PCR measurement of SASP expression upon various treatments of cells as indicated. Data in (g) are shown as mean ± SD and representative of three independent biological replicates. *p* values were calculated by two‐sided *t*‐tests. *p < 0.05; **p < 0.01; ***p < 0.001; ****p < 0.0001.

We next queried the potential mechanisms enabling PQQ to exert an inhibitory role in regulating SASP expression. As cellular senescence usually involves the incidence of DDR, which mediates intracellular signal transduction to functionally support SASP development, we asked about the potential changes of key molecules intimately correlated with the DDR. Upon immunoblot analysis of cell lysates from senescent cells and their proliferating counterparts, we noticed that ATM, a central regulator of DDR, was activated via phosphorylation in response to BLEO treatment, a process that was indeed accompanied by H2AX phosphorylation (Figure 3a). However, these changes remained largely unaffected when cells were treated with PQQ.

Upon exposure to genotoxic stress, proliferating cells typically display an acute stress‐associated phenotype (ASAP), evidenced by rapid ATM activation, nucleus‐to‐cytoplasm translocation and TRAF6‐mediated mono‐ubiquitination prior to TAK1 phosphorylation (Zhang et al. 2018). Our experiments confirmed TAK1 phosphorylation in response to DNA damage treatment, implying its involvement in the activation of downstream events that regulate the SASP. We noticed the activation of intracellular signaling pathways, particularly p38 and Akt/mTOR axes in senescent cells, consistent with the development of their chronic secretory phenotype, the SASP (Figure 3a). However, in the presence of PQQ, these changes were markedly alleviated, although with the exception of ATM activation and TAK1 phosphorylation. These observations imply that PQQ likely targets a molecule positioned downstream of ATM and TAK1, while upstream of p38 and its regulated cascade molecules (Figure 3a). In terms of functional role, this target of PQQ appears to be crucially involved in an early response of cells after genotoxic stress but prior to expression of the full spectrum SASP.

Although the expression of cell division kinase inhibitors (CDKIs), including p53, p21, and p16, was generally upregulated in response to BLEO‐delivered genotoxicity, PQQ treatment did not change the pattern, suggesting the target of PQQ is likely downstream of these CDKIs (Figure 3a). However, we observed a significant upregulation of typical SASP markers, including IL‐6, IL‐8, MMP3, and MMP10, in protein lysates of senescent cells, with the tendency clearly subjected to reversal by PQQ treatment (Figure 3a). Importantly, these changes were largely consistent with alterations at the transcript level as indicated by data from quantitative real‐time PCR (qRT‐PCR) assays (Figure 2c), thus substantiating the inhibitory effect of PQQ on expression of the SASP. Immunoblot data also suggested that PQQ per se does not alter Nrf2 expression, but BLEO caused upregulation of Nrf2. When senescent cells were exposed to PQQ, Nrf2 expression was further elevated, largely in line with the finding that PQQ activates Nrf2 and its correlated signaling (Xue et al. 2024).

To identify the direct molecular target of PQQ, we performed assays of drug affinity responsive target stability (DARTS) combined with liquid chromatography–tandem mass spectrometry (LC/MS–MS). Data from DARTS/LC/MS–MS suggested potential interactions between PQQ and a group of intracellular molecules, including the heat shock protein A8 (HSPA8; Figure 3b,c; Table S2). HSPA8 is a molecular chaperone involved in protein folding, stability maintenance, and the regulation of various cellular processes, including inflammation and autophagy (Xu et al. 2024). Our recent study demonstrated functional involvement of HSPA8 in mediating development of the SASP upon cellular senescence. To substantiate that HSPA8 is a direct target of PQQ, we queried the biophysical properties of the interaction between HSPA8 and PQQ. As a strategy to assess protein thermal stabilization upon ligand binding, a cellular thermal shift assay (CETSA) was conducted in a temperature range from 30°C to 70°C. We noticed decreased stability of HSPA8 upon incubation with PQQ as compared to the vehicle (Figure 3d). In addition, data from surface plasmon resonance (SPR) experiments showed a strong interaction between PQQ and HSPA8 (Figure 3e). To identify the residue(s) of HSPA8 responsible for binding by PQQ, we performed in silico molecular modeling, with the results showing that PQQ can establish direct hydrogen bond connections with HSPA8 via residues Glu268 and Asp366 (Figure 3f). Together, the identification of HSPA8 as a direct target of PQQ supports a novel and essential regulatory mechanism through which PQQ may interfere with HSPA8‐mediated intracellular signaling, including the activation of its downstream pathways that allow signal transduction from the ASAP toward a subsequent culmination of the secretory phenotype, a fully blown SASP.

Since PQQ directly and specifically targets HSPA8, the interactions of HSPA8 with ATM and p38, two molecules that are physically bound by HSPA8 upon cellular senescence, may be affected and result in altered status or activity of ATM and p38. To prove this, we treated senescent cells with VER155008 (VER), a small molecule chemical inhibitor of HSPA8, and measured the SASP expression at the transcription level. The data suggested reduced expression of typical SASP factors (Figure 3g). Furthermore, immunoblot data indicated diminished activation of key SASP regulators, including p38 and mTOR, although the activities of their upstream modulators, such as ATM, remained basically unchanged (Figure S4a). We further determined the effect of PQQ on nuclear translocation of p65 (RelA), a key NF‐κB subunit implicated in multiple cellular processes. Immunoblots indicated that exposure of senescent cells to PQQ blocked the nuclear transportation of p65 (Figure S4b). Results from immunofluorescence staining largely convinced the effect of PQQ on p65 nuclear translocation (Figure S4c). These data suggest that PQQ can prevent the NF‐κB complex from activating during cellular senescence, further substantiating an important role of PQQ in restraining the development of the SASP.

Senescent cells are correlated with elevated oxidative stress, which can be caused by reactive oxygen species (ROS) generated by dysfunctional mitochondria. In response to genotoxicity delivered by BLEO, senescent cells displayed increased ROS production as compared with their normal counterparts (Figure S4c). Although PQQ did not reduce the ROS level in proliferating cells, it significantly restrained ROS generation in senescent cells, consistent with its role as a free radical scavenger. In addition to ROS production, mitochondrial membrane potential (MMP) decline is widely observed in senescent cells and is indicative of mitochondrial dysfunction. To this end, we measured the MMP levels and noticed that PQQ treatment markedly counteracted the decline of MMP, a case observed in senescent cells, thus substantiating its antioxidant benefit in these cells (Figure S4d).

Together, our data suggest that PQQ, a redox cofactor, targets the molecular chaperone HSPA8, disturbing activation of downstream signaling pathways including the axes mediated by p38/Akt/mTOR and the NF‐κB complex, finally resulting in downregulated expression of the SASP. In addition, PQQ reduces ROS production and alleviates MMP decline, both of which are drivers of mitochondrial dysfunction and SASP expression in senescent cells. These findings highlight the therapeutic potential of PQQ, a redox‐active quinone molecule, in regulating senescence‐related phenotypes, particularly the pro‐inflammatory activity of senescent cells, a geroprotective capacity that merits further exploration with in vitro assays and in vivo experiments.

### 2.4 PQQ Restrains Cancer Cell Malignancy Conferred by Senescent Stromal Cells

**FIGURE 4:**
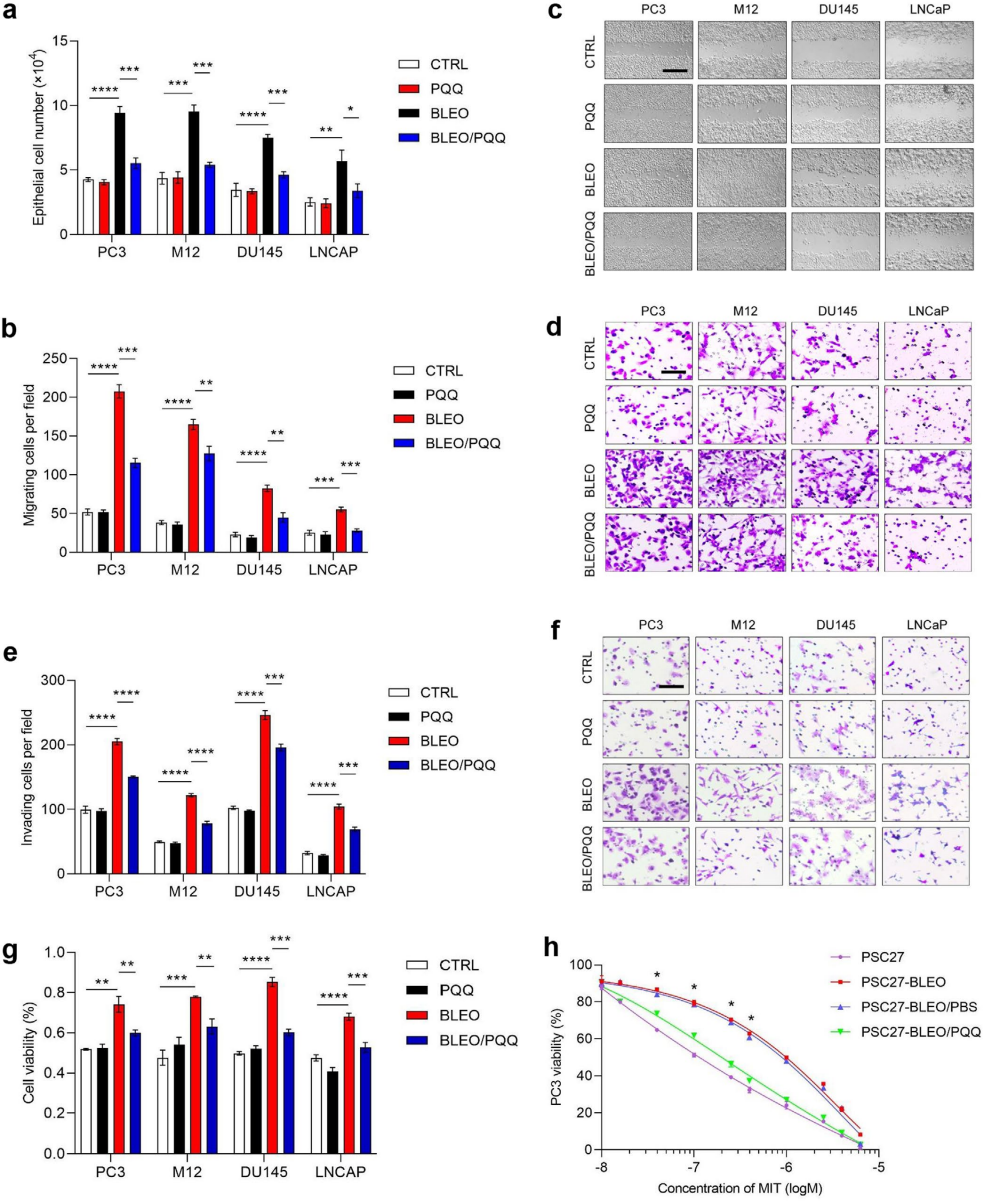
PQQ reduces the malignancy of prostate cancer (PCa) cells conferred by senescent stromal cell‐derived conditioned media. (a–f) PCa cell lines (PC3, M12, DU145, and LNCaP) were cultured for 3 days with conditioned media (CM) from PSC27 stromal sublines and subjected to assays for proliferation (a), migration (b–d) and invasion (e, f). The CM was collected from an equal number of cells *per* condition, with a starting DMEM containing 0.5% FBS to make the CM. Wound healing (c) and crystal violet (d) assays were performed to evaluate the migration capacity of cancer cells, with statistical analysis performed to measure (d). (e, f) Invasion assay to examine the invasiveness of cancer cells, with statistical analysis (e) and representative images (f) shown. (g) Chemoresistance assay of PCa cells cultured with the CM from PSC27 sublines described in (a). Mitoxantrone (MIT) was applied at the concentration of IC50 value predetermined *per* PCa line. (h) Dose–response curves (nonlinear regression/curve fit) plotted from MIT‐based survival assays of PC3 cells cultured with the CM of native PSC27 or BLEO‐induced senescent cells (PSC27‐BLEO) and concurrently treated by a range of concentrations of PQQ. In (c), (d), and (f), scale bars, 50 μm. Data are representative of 3 independent experiments. All *p* values were calculated by two‐sided *t*‐tests. *p < 0.05; **p < 0.01; ***p < 0.001; ****p < 0.0001.

Former studies proved that a full‐spectrum SASP can significantly promote the malignancy of cancer cells by acting as a key driver of tumor progression (Demaria et al. 2017; Zhang et al. 2018). In this study, we chose to determine the impact of PQQ on in vitro behaviors of human prostate cancer (PCa) cells by culturing them with conditioned media (CM) derived from stromal cells. Not surprisingly, CM from senescent PSC27 cells markedly increased the proliferation of PCa lines PC3, M12, DU145, and LNCaP (*p* < 0.01; Figure 4a). Changes in proliferation were largely consistent with elevated migration and invasion activities of cancer cells (Figure 4b–f). However, when stromal cells were exposed to PQQ, the pro‐tumorigenic effects caused by stromal cell‐derived CM were remarkably weakened (Figure 4a–f).

It is noticeable that the viability of cancer cells was significantly enhanced upon treatment with senescent stromal cell‐generated CM but markedly counteracted upon exposure of stromal cells to PQQ (Figure 4g). Although PSC27‐BLEO CM increased the viability of PC3 cells, which were exposed to a chemotherapeutic agent, mitoxantrone (MIT), applied at doses across 0.1–1.0 μM, a dose range close to serum concentrations of MIT delivered to PCa patients in clinical conditions (Costa et al. 2020), PQQ remarkably deprived cancer cells of resistance conferred by senescent stromal cell‐derived CM (Figure 4h). To rule out the potential contribution of increased cell proliferation to the observed resistance of cancer cells, we measured both activities in shortened periods, such as 24 h after experimental initiation. Interestingly, cancer cells showed significantly enhanced resistance to MIT, even when they did not acquire elevated proliferative capacity at the 24th h (Figure S5a,b). However, cancer cell resistance appeared still markedly weakened in the case of PQQ treatment of stromal cells. Altogether, these data suggest that cancer cells hold the potential to develop chemotherapeutic resistance, an activity that is independent of increased proliferative capacity, although subject to reversal by PQQ.

Therefore, targeting the full spectrum of SASP factors with a small molecule compound that has senomorphic capacity, like PQQ, can significantly reduce the malignancy of cancer cells, particularly stromal cell‐conferred gain‐of‐functions, including resistance to chemotherapeutic drugs. These findings provide a solid foundation for further assays, such as developing new therapeutic regimens to enhance anticancer efficacy under in vivo conditions.

### 2.5 Combining Chemotherapy With PQQ Improves Treatment Efficacy in Experimental Mice

Given the remarkable impact of PQQ on the secretory phenotype of senescent cells and subsequent influence on the malignancy of cancer cells, we next investigated its therapeutic potential under in vivo conditions. To this end, we produced tissue recombinants by mixing individual PSC27 sublines with PC3 cells at an optimized ratio of 1:4 prior to subcutaneous implantation into the hind flanks of mice engineered to have severe combined immunodeficiency (SCID). After an 8‐week period, we measured tumor size to evaluate PQQ's therapeutic effect. In relation to tumors comprising PSC27^Naive^ and PC3, xenografts composed of PSC27^SEN^ and PC3 displayed significantly increased sizes, confirming the pro‐tumorigenic role of senescent cells in the microenvironment (Figure S6a). However, pretreatment of PSC27^SEN^ cells with PQQ in culture prior to tissue recombination led to substantially reduced tumor sizes (*p* < 0.001), a phenomenon reminiscent of the consequence of treatment by rapamycin or rutin, each a previously reported natural senomorphic agent that holds the potential to cause a long‐term in vivo effect even after treating senescent human cells in vitro for only once (Laberge et al. 2015; Liu et al. 2024).

**FIGURE 5:**
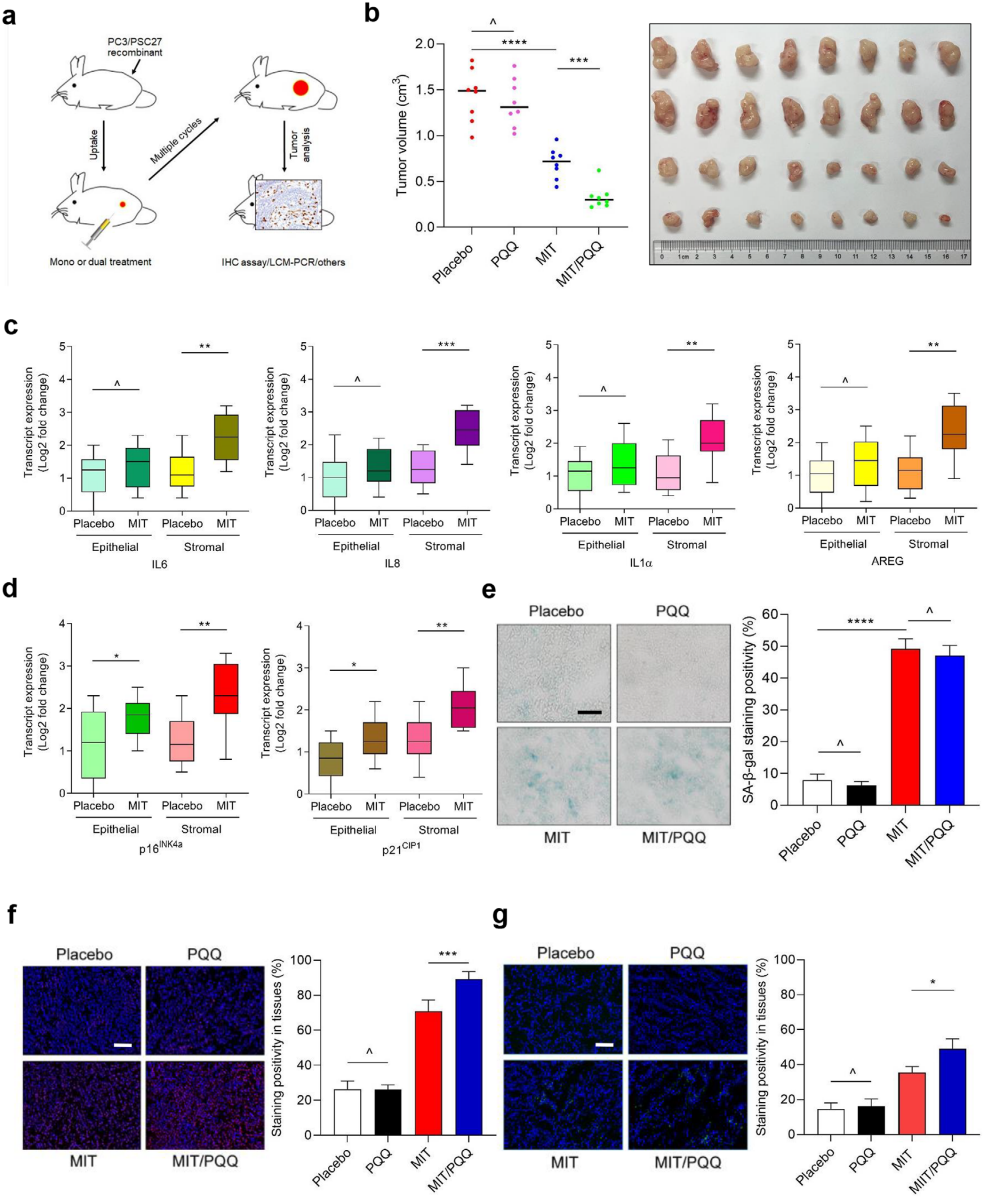
Combination of chemotherapy and PQQ improves therapeutic outcomes in preclinical trials. (a) Schematic representation of the experimental design in severe combined immunodeficient (SCID) mice. Two weeks after subcutaneous implantation of tissue recombinants, animals received either single‐agent or combination therapy administered in metronomic cycles. (b) Statistical comparison of tumor end volumes between treatment groups. PC3 cells were xenografted either alone or together with PSC27 cells to the hind flank of SCID mice, with MIT administered to induce tumor regression. Left, comparative statistic. Right, representative tumor images. The chemotherapeutic agent mitoxantrone (MIT) was used to induce tumor regression. (c) Quantitative measurement of the expression of SASP factors IL‐6, CXCL8, IL‐1α, and AREG at the transcription level. Tissues from animals implanted with both stromal and cancer cells were subject to laser capture microdissection (LCM) isolation, total RNA preparation, and expression assays. (d) Expression analysis of senescence markers including p16^INK4a^ and p21^CIP1^ with tissues from animals as described in (c). (e) Determination of cellular senescence in tumor tissues. Left, representative images of SA‐β‐gal staining in xenografts of each group. Scale bar, 200 μm. Right, comparative statistics. (f, g) Statistical appraisal of DNA damage and apoptosis in the tumor specimens analyzed in (e). Values are presented as a percentage of cells positively stained by immunofluorescence staining with antibodies against γH2AX or caspase 3 (cleaved). Biopsies of placebo‐treated animals served as negative controls for MIT‐treated mice. Scale bars, 50 μm. Data in (b–g) are representative of three independent experiments. All *p* values were calculated by Student's *t*‐tests. ^*p* > 0.05; *p < 0.05; **p < 0.01; ***p < 0.001; ****p < 0.0001.

To closely simulate chemotherapeutic settings observed in clinical oncology, we generated a preclinical procedure to incorporate genotoxic drugs and/or PQQ (Figures 5a and S6b). Two weeks after implantation of human cells, when stable tumor uptake was observed in host animals, we delivered a single dose of placebo or MIT on the 1st day of the 3rd, 5th, and 7th weeks until completion of the 8‐week regimen. In contrast to the placebo, MIT treatment caused a significant reduction in tumor volume, thus validating its effectiveness as a cytotoxic drug (Figure 5b). We observed a significant upregulation of SASP factors, such as IL‐6, IL‐8, IL‐1α, AREG, MMP3, BMP6, ANGPTL4, and SPINK1, a change that was concurrent with the expression of senescence markers, including CDKIs p16^INK4a^ and p21^CIP1^ I n xenografts comprising PC3 and PSC27 cells, implying the incidence of in vivo senescence and development of the SASP in response to treatment by a chemotherapeutic agent (Figures 5c,d and S6c). It is interesting that some SASP factors, such as GM‐CSF, alongside the canonical senescence markers p16^INK4a^ and p21^CIP1^, were induced by MIT in both stromal cells and their cancer cell counterparts, suggesting that chemotherapy induced broad in vivo senescence, although the SASP profile appeared different between these two cell subpopulations (Figures 5d and S6c). Results from SA‐β‐gal staining indicated senescence induction at the tissue level after MIT treatment, in sharp contrast to PQQ, which indeed neither promoted nor inhibited senescence in the tumor microenvironment (Figure 5e), a pattern indeed basically consistent with the data from in vitro assays (Figure 2a).

To investigate the mechanisms underlying cancer resistance conferred by the SASP, we analyzed tumors excised from animals 7 days after the start of treatment, a timepoint prior to the emergence of resistant cell colonies. PQQ per se neither caused a typical DDR nor induced significant cell death in PC3/PSC27 tumors, as indicated by data from immunofluorescence staining against γH2AX or caspase 3 cleavage, indicating a limited response of these xenografts when animals were subject to PQQ treatment alone (Figure 5f,g). However, once combined with MIT, PQQ further increased the percentage of apoptotic cells, implying increased cytotoxicity upon co‐treatment with both MIT and PQQ, with the in vivo apoptosis pattern generally in line with the tumor regression profile upon treatment by the individual agents.

Given the efficacy of PQQ in improving treatment outcomes once combined with conventional chemotherapy, we next asked about the safety and feasibility of the regimen. To address this question, we performed a series of routine pathophysiological analyses. The resulting data indicated that either single or combinational treatment was tolerated by animals, as evidenced by the absence of significant body weight fluctuation throughout the therapeutic period (Figure S6d). Furthermore, no significant perturbations in the serum levels of creatinine, urea, or activities of alkaline phosphatase (ALP) and alanine aminotransferase (ALT), the latter two measurements indicative of liver metabolic activities, were observed (Figure S6e). Therefore, our data consistently support that the combination of PQQ, a senomorphic agent, with classical chemotherapy holds the potential to enhance tumor response but without increasing the risk of triggering systemic cytotoxicity.

### 2.6 PQQ Alleviates Pathological Changes in Naturally Aged Mice Without Reversing Tissue Senescence

**FIGURE 6:**
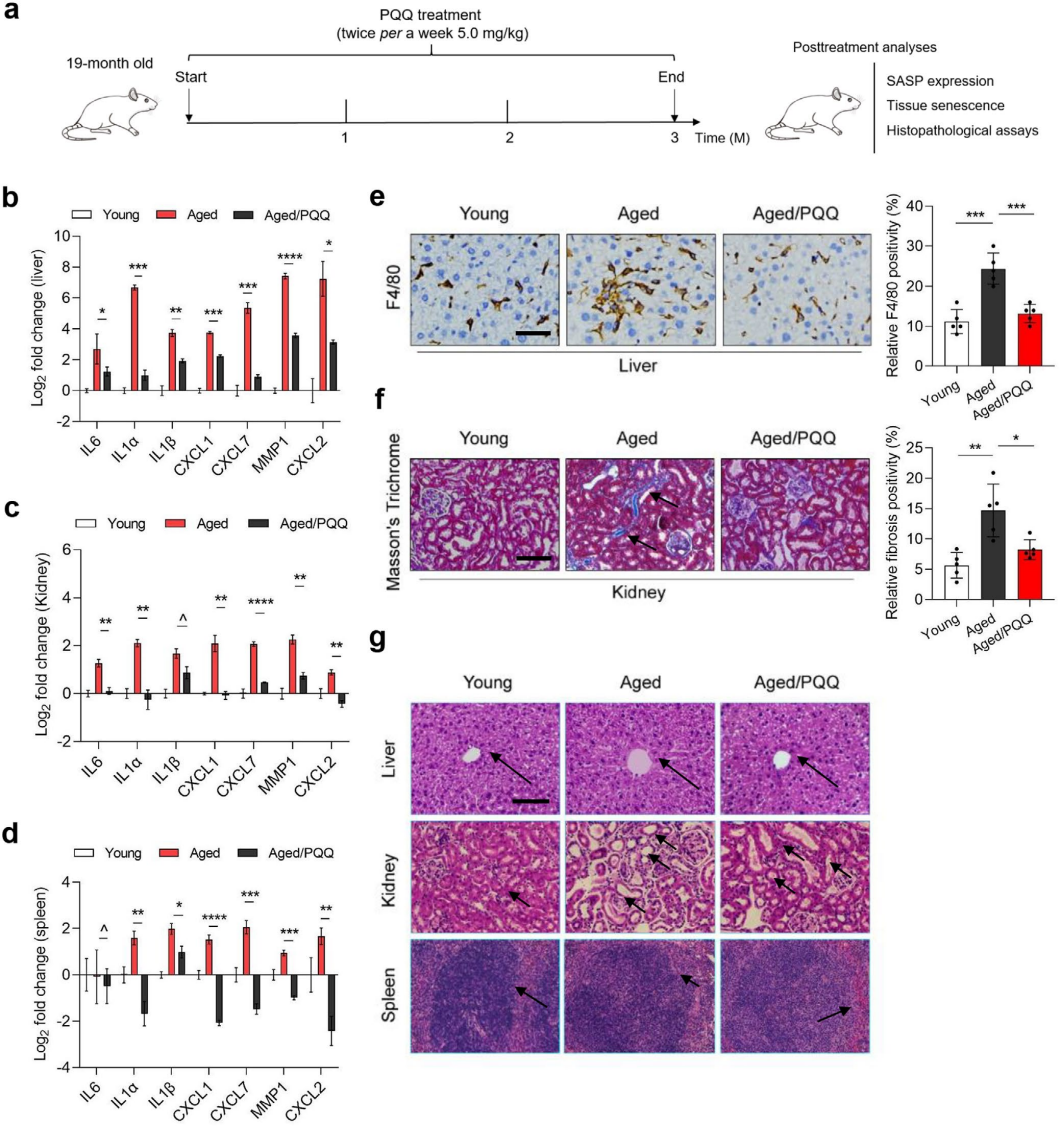
PQQ alleviates age‐related pathological changes in solid organs of naturally aged mice without affecting tissue‐level senescence. (a) Schematic representation of treatment schedule for naturally aged mice. (b–d) Quantitative measurement of SASP expression at transcription level in liver, kidney and spleen, tissues. (e) IHC analysis of F4/80 expression in liver tissues. Left, representative images of F4/80 staining in xenografts of each group. Scale bar, 20 μm. Right, comparative statistics. (f) Masson's trichrome staining of kidney tissue. Left, representative images of Masson's trichrome staining in xenografts of each group. Arrows, collagen fibers. Scale bar, 20 μm. Right, comparative statistics. (g) Hematoxylin and eosin (H&E) staining was performed on tissue sections from liver, kidney, and spleen to evaluate pathological features associated with aging. Representative images show the morphology of each organ, highlighting the characteristic alterations indicative of aging, such as tissue degeneration and fibrosis. Arrows in liver and kidney denote areas that exhibit tissue degeneration. Arrows in spleen denote boundaries between the red and white pulp. Scale bar, 50 μm. Data are shown as mean ± SD and representative of three independent experiments (b–f). *p* values were calculated by Student's *t*‐test. ^*p* > 0.05. *p < 0.05. **p < 0.01; ***p < 0.001; ****p < 0.0001.

As PQQ generates a prominent inhibitory effect on SASP expression, we further interrogated its therapeutic potential in modulating aging. To answer this, we chose to employ experimental mice at 19 months of age. Animals received a 3‐month period of treatment by PQQ, which was administered at a dose of 5.0 mg/kg twice *per* week. At the end of the therapeutic regimen, we assessed the expression of SASP factors, senescence induction, and histopathological profile of different organ types, a set of measurements that help appropriately evaluate the effect of PQQ in geroprotective interventions (Figure 6a).

Quantitative transcription‐based analysis (qRT‐PCR) indicated a significant reduction of most SASP factors in the tissues of the liver, kidney, and spleen of PQQ‐treated mice (Figure 6b–d). Liver aging is accompanied by remarkable changes in Kupffer cells, a group of liver‐resident macrophages. These cells show reduced phagocytic activity and increased secretion of pro‐inflammatory cytokines such as IL‐6 and IL‐1β, which contribute to chronic inflammation and fibrosis, two closely related chronic liver pathologies (Taru et al. 2024). Results from immunohistochemistry staining revealed an increase in the percentage of F4/80‐positive macrophages in the liver of aged mice, indicating a shift toward pro‐inflammatory M1 macrophages (Figures 6e and S7a,b), which can exacerbate local inflammation (Wei et al. 2025). Of note, PQQ treatment significantly reduced the abundance of M1 macrophages in the liver of aged mice, suggesting that PQQ exerted an anti‐inflammatory property. PQQ appeared to have promoted a shift from M1 to the anti‐inflammatory M2 phenotype (Figures 6e and S7a,b), a change that may help alleviate liver inflammation, reduce fibrosis, and restore immune balance in the aged liver. These results imply the potential of PQQ as a therapeutic agent to mitigate liver fibrosis and reduce inflammation levels increased with age, thus improving liver condition.

Results from Masson's trichrome staining with kidney tissues suggested increased collagen deposition and extracellular matrix accumulation, changes that are indicative of fibrosis (Figure 6f; Bernhardt et al. 2023). The fibrotic progression is accompanied by chronic inflammation, oxidative stress, and cellular senescence, which ultimately impair kidney function. However, PQQ treatment appeared to have attenuated renal fibrosis, as evidenced by reduced collagen deposition and improved extracellular matrix remodeling (Figures 6f and S7c,d). We observed normalization of renal tubular and glomerular structures and reduced tissue remodeling (Figure 6g). The data suggest that the geroprotective properties of PQQ help protect renal function by preventing the incidence of fibrosis in aged mice. PQQ treatment also generated beneficial effects such as partially restored spleen structure and improved spleen tissue morphology (Figure 6g). Specifically, in young animals the white pulp was uniform in size and mostly regular in shape, with clear boundaries between the red and white pulp. In contrast, in aged mice, the boundaries between the red and white pulp seemed to be blurred, with white pulp sparsely arranged and the gaps widened. Upon PQQ treatment, the contours of the white pulp became largely restored and appeared to be more distinct.

Although PQQ treatment improved histopathological features in multiple organs, however, it did not reverse cellular senescence, as indicated by staining positivity of the senescence marker SA‐β‐gal in the liver, kidney, and spleen (Figure S8a–d). The data suggest that PQQ does not reverse senescence at the tissue level but rather mitigates age‐related pathological changes via its geroprotective function, which is closely correlated with its anti‐inflammatory property. To further extend this study, we measured plasma levels of creatinine, aspartate aminotransferase, alanine aminotransferase, blood urea nitrogen, and triglyceride. The data showed that old animals had generally elevated levels of these biochemical indices relative to their young counterparts, but most of these parameters were subject to reversal by PQQ administration (Figure S8e–i). Thus, PQQ treatment helps achieve functional improvement of the liver and kidney in aged animals.

In summary, our study demonstrates that PQQ alleviates age‐related tissue dysfunction and chronic inflammation in solid organs, including the liver, kidney, and spleen, without reversing the fundamental process of senescence per se. Thus, these findings suggest that PQQ holds a promising potential as a potent senomorphic agent by targeting the pro‐inflammatory phenotype, mainly the SASP, of senescent cells to restrain pathological alterations associated with chronological aging.

## 3 Discussion

PQQ is an aromatic and water‐soluble quinone, with chemical properties largely equivalent to the combination of the chemical features of riboflavin, ascorbic acid, and pyridoxal 5‐phosphate into one molecule (Jonscher et al. 2021). Besides acting as a free radical scavenger, PQQ protects against ROS‐mediated oxidative stress and is well known for its potent antioxidant capacity (Yan et al. 2024). PQQ has received approval from the Food and Drug Administration (FDA) as a dietary supplement, making it an attractive candidate for intervention of aging and age‐related conditions. In this study, we investigated the therapeutic potential of PQQ as a novel senotherapeutic agent. The data suggest that although PQQ does not eliminate senescent cells as a senolytic compound, it hampers the SASP development and mitigates the pro‐inflammatory effect of senescent cells. As senotherapeutics hold the potential to address root causes of age‐related pathologies and thus open new avenues to prevent, delay, and treat multimorbidities (McHugh et al. 2024), we explored the efficacy of PQQ in mitigating age‐related diseases such as cancer and alleviating age‐related changes of organ structure. The study established the potential of PQQ in future development as an effective geroprotective agent (Figure S8).

Previous studies reported that PQQ can protect against IL‐1β‐induced matrix degradation and ROS accumulation while decreasing the expression of certain SASP factors in human nucleus pulposus cells (NPCs) in vitro (Xue et al. 2024). Specifically, treatment with PQQ reversed IL‐1β‐induced changes in senescence marker p16^INK4a^, extracellular matrix proteins including MMP13 and collagen II, and cell proliferation in NPCs, wherein PQQ disrupts the Keap1‐Nrf2 complex and activates Nrf2‐ARE signaling, causing Wnt5a upregulation and preventing aging‐related IVDD (Xue et al. 2024). Despite these important functions, the effect of PQQ on senescent cells, particularly their pro‐inflammatory activity reflected by expression and secretion of SASP factors, remains unclear. Moreover, both the direct target and underlying mechanism of PQQ in targeting senescent cells warrant further characterization, ideally with cell lineages that are more universal across solid organs, such as fibroblasts.

PQQ exhibits potent antioxidant properties, mainly by protecting the mitochondria against oxidative stress‐mediated lipid peroxidation and protein carbonyl formation, and minimizes damage to the respiratory chain (Gao et al. 2022b). In our study, we found that PQQ can markedly decrease intracellular ROS levels and antagonize MMP decline in senescent cells, but not in their proliferating counterparts, suggesting the potent anti‐senescence potential of PQQ. Although PQQ effectively downregulated expression of the SASP, cellular senescence per se remained basically unaffected, further substantiating its senomorphic nature. While it was reported that PQQ treatment can reverse IL‐1β‐induced upregulation of senescence markers including p16^INK4a^, the phenomenon may be limited to the specific cell line examined, such as NPC (Xue et al. 2024). In this study, we investigated the capacity of PQQ in restraining the SASP expression in several cell lineages of stromal origin, including fibroblasts, endothelial cells, and mesenchymal stem cells. The data suggest that PQQ can exert its senomorphic function in a wide range with an optimal concentration such as 100 μM in vitro, approaching that of other natural agents such as rutin (Liu et al. 2024). Although higher concentrations may inhibit the SASP even more significantly, they may also affect the status or survival of proliferating cells, and an appropriate concentration is supposed to differentiate between senescent cells and their normal counterparts.

The protein HSPA8 belongs to the heat shock protein 70 family, which comprises both heat‐inducible and constitutively expressed members. Often referred to as a heat shock cognate protein, HSPA8 functions as a chaperone and binds to nascent polypeptides to facilitate correct folding. Former studies indicated that HSPA8 can interact with aldehyde dehydrogenase 2 (ALDH2) to regulate fibroblast senescence after oxygen–glucose deprivation, providing an option to effectively intervene in fibroblast senescence after myocardial infarction (Hui et al. 2023). Downregulation of MAGI2‐AS3, a long noncoding RNA (lncRNA) involved in redox regulation, reduces the hydrogen peroxide content through stabilizing the HSPA8 protein via inhibition of proteasome degradation of HSPA8 and suppresses cellular senescence, wherein MAGI2‐AS3 interacts with the C‐terminal domain of HSPA8 (Zhang et al. 2022). However, the relevance of HSPA8 to the pro‐inflammatory activity of senescent cells remains hitherto underexplored. Our recent study focusing on the effect of a natural flavonoid, apigenin, on senescent cells disclosed the involvement of HSPA8 in mediating intracellular signal transduction of the SASP, although downstream of PRDX6. Specifically, treatment of senescent cells with apigenin can significantly reduce SASP expression in various cell lineages of stromal origin. Our proteomic data suggested that HSPA8 may be a direct target of PQQ, although exposure of senescent cells to PQQ cannot completely diminish SASP expression. As the results imply that molecular targets of PQQ are not limited to HSPA8, the identity of the remaining molecular target(s) of PQQ deserves further investigation by future efforts.

Given the potent antioxidant property, as evidenced by the capacity of free radical scavenging and oxidative stress reduction, PQQ holds a promising potential to be applied for both the treatment and prevention of age‐related diseases (Cheng et al. 2021). In addition to the therapeutic value of PQQ in various neurodegenerative diseases by playing a neuroprotective role, PQQ can effectively control tumor growth, wherein it acts as an anti‐tumor agent with minimal or no toxicity to normal cells by activating mitochondrial‐dependent apoptotic pathways following PQQ treatment (Wu et al. 2018). In our study, the dose of PQQ chosen to treat experimental mice carrying tumor xenografts did not show significant effects on tumor progression, while a combination of PQQ and chemotherapy did. In contrast to the condition that allowed the mice to be given a daily abdominal injection of 50.0 mg/kg PQQ in the case of chondrosarcoma treatment (Wu et al. 2018), our dose was limited to 5.0 mg/kg. While the varying doses contribute to the differential effects observed between studies, our study highlights that PQQ holds the capacity to improve therapeutic outcome by acting as an effective senomorphic agent, rather than by targeting cancer cells per se in the anticancer regimen.

Due to the antioxidant property and the ability to maintain mitochondrial activity, studies have suggested that PQQ may play a role in extending lifespan and slowing down the aging process (Mohamad Ishak et al. 2024). Our study suggests that PQQ alleviates age‐related tissue dysfunction and chronic inflammation across multiple organs, including the liver, kidney, and spleen, but without reversing the fundamental process of cellular senescence. This protective effect of PQQ may be correlated with its capacity to mitigate age‐related mitochondrial dysfunction, thus maintaining optimal metabolic activities in these organs. Despite the preservation of senescent cells in tissue microenvironments, our findings suggest a promising potential of PQQ as an effective senomorphic agent by alleviating the pathological conditions of aging. However, since senescent cells, particularly those highly expressing p16, are able to play beneficial roles in certain situations, such as tissue repair and wound healing (Gasek et al. 2025), targeting the SASP with PQQ as a senomorphic agent may interfere with these processes. Thus, caution should be exercised to minimize potential side effects when using PQQ to mitigate age‐related conditions.

Macromolecule damage‐induced ROS accumulation and cellular senescence have been recognized as the key driving forces in the pathogenesis of several age‐related diseases, including cancers. A number of naturally occurring compounds, including those with a polyphenolic structure, can extend the lifespan and improve the healthspan of organisms (Russo et al. 2020). Due to their inherent phytochemical properties, understanding their targets and functional mechanisms could be challenging and complicated. As a water‐soluble and vitamin‐like natural compound, PQQ does not belong to the polyphenol family, but it has essential physiological functions such as anti‐oxidation, anti‐inflammation, anti‐aging, and immunity enhancement (Cordell and Daley 2022). However, due to the lack of in‐depth understanding of PQQ biosynthesis and regulation, inefficient PQQ production limits its wide application. To date, there remains an urgent need to develop high‐yielding strains and/or microbial species with optimized biosynthetic pathways to improve PQQ synthesis efficiency (Gao et al. 2023). Despite the fact that the discovery and validation of PQQ, a safe and effective senomorphic agent, contribute to the expanding arsenal of senotherapeutics, laying the baseline for developing innovative and safe therapeutics to intervene in age‐related pathologies.

Together, the functional property of PQQ in targeting senescent cells while preserving their viability in age‐related conditions warrants further investigation. Future efforts are necessary to determine long‐term outcomes and the therapeutic potential of PQQ for intervention in geriatric syndromes in clinical settings, particularly its capacity to extend the lifespan and healthspan of humans by providing a series of geroprotective effects for those in advanced life stages.

## 4 Methods

### 4.1 Cell Culture

Primary normal human prostate stromal cell line PSC27 was a courteous gift from Dr. Peter Nelson (Fred Hutchinson Cancer Center) and maintained in stromal complete medium as described previously (Sun et al. 2012). Prostate cancer epithelial cell lines PC3, DU145, and LNCaP (ATCC) were routinely cultured with RPMI 1640 (supplemented with 10% FBS). Prostate cancer epithelial line M12 was kindly provided by Dr. Stephen Plymate (University of Washington), which was originally derived from the benign line BPH1 but phenotypically neoplastic and metastatic (Bae et al. 1998). The MEFs were cultured in high glucose DMEM (supplemented with 10% FBS). HUVECs were cultured in endothelial cell mesenchymal stem cell growth medium 2 (PromoCell). All lines were routinely tested for mycoplasma contamination and authenticated with STR assays.

### 4.2 Cell Treatments

Stromal cells were grown until 80% confluent (CTRL) and treated with 50 μg/mL bleomycin (BLEO) to induce senescence. After treatment, the cells were rinsed briefly with PBS and allowed to stay for 7–10 days before being subjected to the performance of various examinations. Senotherapeutic agents screened in this study were provided by BY‐HEALTH (https://www.by‐health.com). To search for potential senolytics, natural candidates (46 in the NMA library) were tested, each at 3 μg/mL, on the survival of 5.0 × 10^3^ CTRL and BLEO cells for 3 days. Viable cells were counted with brightfield microscopy. Subsequently, evaluation of the effects of remaining agents (41 in the library, each applied at 1 μg/mL) was performed to test the efficacy of SASP inhibition (senomorphics) by examining the expression level of IL‐1α, a typical SASP factor. IL‐1α expression was assessed by quantitative RT‐PCR to determine its expression level in cells treated by various agents, with cells in 6‐well plates collected to determine IL‐1α expression. The assays allowed excellent separation between positive and negative controls and had low signal variance (*Z*‐factor = 0.85). For those with the potential to act as effective and safe senomorphics, further assessments were conducted. The small molecule compound PQQ was applied in the range of 20–100 μM, with 100 μM determined to be an optimal concentration for senomorphic activity. The small molecule inhibitor VER155008 was used at 4.0 μΜ to treat senescent cells in culture before cell collection for lysis and expression analysis.

### 4.3 SA‐β‐Gal Staining

For SA‐β‐galactosidase (SA‐β‐gal) staining, cells were seeded in the 6‐well plates and fixed with 4% paraformaldehyde for 15 min at room temperature before being washed with PBS. Cells were incubated with SA‐β‐gal solution (pH 6.0; Cell Signaling Technology) at 37°C overnight. The percentage of senescent cells was determined by counting at least 200 cells from multiple images per condition. For tissue staining, freshly dissected samples were embedded in OCT and sectioned (8–10 μm). Frozen sections were fixed with 4% paraformaldehyde for 15 min at room temperature and washed with PBS (pH 6.0). Sections were stained overnight with SA‐β‐gal staining buffer at 37°C, with SA‐β‐gal‐positive cells quantified by ImageJ (v1.51, NIH).

### 4.4 Immunoblot and Immunofluorescence Analysis

Whole cell lysates were prepared using RIPA lysis buffer supplemented with protease/phosphatase inhibitor cocktail (Biomake). Nitrocellulose membranes were incubated overnight at 4°C with primary antibodies, and HRP‐conjugated goat anti‐mouse or ‐rabbit served as secondary antibodies (Vazyme). For immunofluorescence, cells on coverslides coated with poly‐D‐Lysine were washed with PBS and fixed with 4% paraformaldehyde for 15 min at room temperature. The slides were permeabilized and blocked in blocking buffer (0.01% Triton X‐100 in TBS/1% BSA) at 37°C for 2 h. Primary antibodies were incubated at 4°C overnight, followed by PBST washing. Secondary antibodies were applied at room temperature for 1 h, followed by PBST washing. After staining, cells were covered with the anti‐fluorescence quencher that contains DAPI solution. Images were captured with fluorescence microscopy. Antibody information is provided in Table S3.

### 4.5 RNA Extraction and Real‐Time Quantitative PCR

Total RNA was extracted from cultured cells or animal tissues using TRIZOL (Magen, R4801‐02). RNA was reverse transcribed to cDNA using TransScript First‐Strand cDNA Synthesis SuperMix (Tiangen) following the manufacturer's instructions. RT‐qPCR was performed using Hieff qPCR SYBR Green Master Mix (YEASEN) on an ABI QuantStudio7 Flex (ABI Q7). Data were analyzed using the 2 (−ΔΔCt) method. RPL13A or GAPDH was used as an internal control to normalize the expression of target genes. Relevant primer sequences are listed in Table S4.

### 4.6 DARTS Assays

DARTS is a label‐free method, as it does not require chemical modifications of the protein or drug. It is based on the concept that ligand‐bound proteins show altered stability against proteolysis compared to ligand‐unbound proteins. DARTS assays were performed to measure the interaction stability of the small molecule PQQ with its target(s) and performed according to a previously reported protocol (Pai et al. 2015). PSC27 cells were lysed with IP lysate buffer on ice with protease and phosphatase inhibitors before being centrifuged at 4°C and quantified by the BCA method. Lysates were allowed to rapidly warm up to room temperature, and DMSO or PQQ (100 μM) was added to the lysates for 1 h incubation in a rotator. Samples were digested by pronase (1:300 (w/w), 10 μg/mL) for 30 min at room temperature. Digestion was stopped by 0.5 M EDTA (pH 8.0), with samples boiled in loading buffer for SDS‐PAGE and immunoblot evaluation or directly subjected to MS analysis for proteomic profiling. The resultant proteomic data of DARTS were deposited to the ProteomeXchange Consortium (http://proteomecentral.proteomexchange.org) through the iProX partner repository with a unique dataset identifier PXD062905.

### 4.7 Cellular Thermal Shift Assay (CETSA)

To determine whether PQQ targets the HSPA8 protein, CETSA‐immunoblot experiments were performed. CETSA is used to examine the binding efficiency of a drug and protein. Briefly, PSC27 cells in culture were trypsinized and precipitated by centrifugation, lysed on ice with RIPA buffer containing a protease inhibitor cocktail, and centrifuged at 14,000 × g at 4°C for 20 min. Each lysate was then equally aliquoted into several parts in Eppendorf tubes and incubated with PQQ at room temperature for 1 h. Samples were heated for 3 min under different temperatures (30°C–70°C). The precipitated proteins were separated from the soluble fraction by centrifugation and boiled at 95°C for 10 min. Samples were subsequently used for immunoblot analysis.

### 4.8 Molecular Modeling of PQQ Binding to HSPA8

The amino acid sequence of PQQ (code: P11142) was acquired from UniProt. The X‐ray diffraction microscopy structure of HSPA8 (PDB ID: 6ZYJ) was acquired from RCSB Protein Data Bank (PDB, https://www.rcsb.org/). The water molecules farther than 4.5 Å from the receptor were removed. The structures of PQQ were obtained from PubChem (https://pubchem.ncbi.nlm.nih.gov/), while the ligand–receptor interaction was docked and analyzed with the ‘Triangle Matcher’ placement method by the Molecular Operating Environment (MOE) software (2019.01.02), using the MMFF94 force field with the gradient convergence set to 0.1 kcal/mol. All protein structures were prepared by the built‐in MOE structure preparation and Protonate3D software tools using the default parameters. The binding site of each protein was generated by the SiteFinder module in MOE. All conformations per ligand were scored using the ‘London dG’ scoring function, submitted to a refinement step based on molecular mechanics, and rescored with the ‘GBVI/WSA dG’ scoring function, a force field‐based scoring function that determines the binding free energy (kcal/mol) of the ligand from a given pose. Ultimately, the binding mode with the best docking score was selected for further analysis. The docking score denotes the affinity between the protein receptor and the docking ligand. The lower docking score indicates the better affinity (Kalathiya et al. 2019). The binding sites of ATP were determined to be located at residues 12–15, 7, 202–204, 268–275, and 339–342. The length of HSPA8 was selected at 5–384 (not full length). This protein has no full‐length crystal or cryoEM structure, except as predicted by Alphafold.

### 4.9 SPR‐Based Binding Affinity Assays

We performed an SPR assay to evaluate the binding affinity of salvianolic acid A/B/E and HSPA8 on a BIAcore T200. We adjusted the pH to 5.0, 4.5, and 4.0 to measure the binding affinity under several pH environments. HSPA8 protein (purchased from MedChemExpress (https://www.medvhemexpress.cn/)) was diluted in sodium acetate solution (pH 4.0) to a final concentration of 10 μg/mL. HSPA8 protein was immobilized covalently on a CM5 sensor chip (GE Healthcare) to reach target immobilization densities of ~5000 resonance units (RU). Then, immobilized HSPA8 protein was applied to capture salvianolic acid A/B/E. The running buffer consisted of PBS‐P (10 mM sodium phosphate, 150 mM NaCl, 0.05% surfactant P20, pH 7.3) and 5% DMSO. Experiments were carried out at 25°C. A series of concentrations (125, 62.5, 31.25, 15.5, 7.8, 3.9, 1.95, and 0.97 μM) of salvianolic acid A/B/E, diluted from the highest concentration, were analyzed at a flow rate of 30 μL min^−1^ in each binding cycle with a contact time of 60 s and a dissociation time of 60 s. A blank immobilization was performed on one channel surface of the chip to calibrate the binding response. Association and dissociation constants were obtained using the Biacore T200 Evaluation software (v.3.0 GE Healthcare). Data was exported to Prism 9 for globally fitting the final curves using the steady‐state fitting model. Each assay was repeated twice.

### 4.10 Measurement of Intracellular ROS

The level of intracellular ROS was determined with a ROS Assay Kit (Beyotime), which employs dichloro‐dihydro‐fluorescein diacetate (DCFH‐DA) as a probe. Briefly, cells were cultured in 6‐well plates for 24 h at 37°C before being washed twice with serum‐free medium. Medium containing 10 μM DCFH‐DA was added. Cells were then incubated for 20 min at 37°C, with light avoided during incubation. After incubation, the cells were washed thrice with serum‐free medium, then observed and photographed using a fluorescence microscope (Nikon). The fluorescence intensity was measured using ImageJ.

### 4.11 Bulk RNA‐Seq Library Preparation, Sequencing, and Analyses

Bulk RNA‐seq was performed on senescent PSC27 cells cultured in regular DMEM or DMEM containing PQQ (100 μM) for 3 consecutive days. Total RNA was extracted using Trizol reagent (Invitrogen) following the manufacturer's procedure. RNA amount and purity *per* sample were measured using NanoDrop 2000 with an OD260/280 = 1.6–1.8. RNA integrity was assessed by Agilent 2100 (Agilent Technologies). All samples had a RIN number of > 8.0. RNA‐seq libraries were prepared following the manufacturer's instructions, with sequencing performed on an Illumina NovaSeq 6000 following the vendor's instruction protocol and signals quantified by the software package RSEM (https://deweylab.github.io/RSEM/).

Quality control of raw reads was performed by Trimmomatic (v.0.39) software (Bolger et al. 2014). The sequencing read quality was verified using FastQC (v.0.11.9; http://www.bioinformatics.babraham.ac.uk/projects/fastqc, 2010). Raw reads were processed with fastp (v.0.22.0; Chen, Zhou, et al. 2018). FASTQ files were aligned to the human genome (GRCh38.p14; Genome Reference Consortium Human Build 38; INSDC Assembly GCA_000001405.28, 12.2013) using HISAT2 (v.2.1.0; Kim et al. 2019). Counts of reads and fragments per kilobase of exon per million (FPKM) were calculated using StringTie (v.2.1.3b; Pertea et al. 2015). Genes of significantly changed expression were defined by a false discovery rate (FDR)‐corrected *p* value < 0.05. Only Ensembl genes of status “known” and biotype “coding” were used for downstream analysis. Raw data of RNA‐seq were deposited in the NCBI Gene Expression Omnibus (GEO) database under the accession code GSE264316. To generate RNA‐seq heatmaps, for each gene, the FPKM value was calculated based on aligned reads, using Cuff links. *Z*‐scores were generated from FPKMs. Hierarchical clustering was performed using the R package heatmap.2 and the distfun = “pearson” and hclustfun = “average”.

### 4.12 In Vitro Cell Phenotypic Characterization

For proliferation assays of cancer cells, 2 × 10^4^ cells were dispensed into 6‐well plates and co‐cultured with conditioned medium (CM) from stromal cells. Three days later, cells were digested and counted with a hemacytometer. For migration assays, cells were added to the top chambers of transwells (8 μm pore), while stromal CM was given to the bottom. Migrating cells in the bottom chambers were stained by 0.4% crystal violet 12–24 h later, with samples examined with an Zeiss Axio Observer A1 inverted microscope. Invasion assays were performed similarly to migration experiments, except that transwells were coated with basement membrane matrix (phenol red free, Corning). Alternatively, cancer cells were subjected to wound healing assays conducted with 6‐well plates, with healing patterns graphed with a brightfield microscope. For chemoresistance assays, cancer cells were incubated with stromal CM, with the chemotherapeutic agent MIT provided in wells for 3 days at each cell line's IC50, a value experimentally predetermined. Cell viability was assayed by a CCK‐8 kit, with the absorbance at 450 nm measured using a microplate reader.

### 4.13 Histology and Immunohistochemistry

Mouse tissue specimens were fixed overnight in 10% neutral‐buffered formalin and processed for paraffin embedding. Standard staining with hematoxylin/eosin was performed on sections of 5–8 μm thickness cut from each specimen block. For immunohistochemistry, tissue sections were deparaffinized and incubated in citrate buffer at 95°C for 40 min for antigen retrieval before being incubated with primary antibodies (e.g., anti‐cleaved Caspase 3, 1:1000) overnight at 4°C. After 3 washes with PBS, tissue sections were incubated with a biotinylated secondary antibody (1:200 dilution, Vector Laboratories) for 1 h at room temperature, then washed thrice, after which streptavidin‐horseradish peroxidase (HRP) conjugates (Vector Laboratories) were added and the slides were incubated for 45 min. DAB solution (Vector Laboratories) was then added, and the slides were counterstained with hematoxylin.

### 4.14 Experimental Animals and Chemotherapeutic Studies

All animals were housed in a specific pathogen‐free (SPF) facility. NOD/SCID mice (Shanghai Model Organisms) aged approximately 6 weeks (average body weight of ~20 g) were used for the study. Each experimental group consisted of 8 mice. Xenografts were subcutaneously established on the hind flanks under isoflurane anesthesia. Prior to implantation, stromal cells (PSC27) and cancer cells (PC3) were combined at a ratio of 1:4, with 250,000 stromal cells admixed with 1,000,000 cancer cells to form tissue recombinants. Mice were sacrificed 2–8 weeks after tumor xenografting based on tumor burden or specific experimental requirements. Tumor growth was monitored weekly, and tumor volume (*v*) was calculated using the formula *v* = (π/6) × ((*l* + *w* + *h*)/3)^3^, where *l*, *w*, and *h* represent tumor length, width, and height, respectively (Chen, Long, et al. 2018; Zhang et al. 2018). Freshly excised tumors were either snap‐frozen or fixed for FFPE sample preparation. The resulting sections were processed for hematoxylin/eosin staining or immunohistochemical (IHC) analysis against specific antigens.

For chemoresistance assessment, animals received subcutaneous implantation of tissue recombinants as described above and were given standard laboratory diets for 2 weeks to allow tumor uptake and growth initiation. Starting from the 3rd week (tumors reaching 4–8 mm in diameter), MIT (0.2 mg/kg doses), the senomorphic agent PQQ (5.0 mg/kg doses, 200 μL/dose), or vehicle controls were administered by intraperitoneal injection (therapeutic agents via i.p. route) on the 1st day of the 3rd, 5th, and 7th weeks, respectively. Upon completion of the 8‐week therapeutic regimen, animals were sacrificed, with tumor volumes recorded and tissues processed for histological evaluation.

At the end of chemotherapy and/or targeted treatment, animals were anesthetized, with peripheral blood gathered via cardiac puncture. Blood was transferred into a 1.5 mL Eppendorf tube and kept on ice for 45 min, followed by centrifugation at 9000 × *g* for 10 min at 4°C. Clear supernatants containing serum were collected and transferred into a sterile 1.5 mL Eppendorf tube. All serum markers were measured using dry‐slide technology on the IDEXX VetTest 8008 chemistry analyzer (IDEXX). About 50 μL of the serum sample was loaded on the VetTest pipette tip before securely fitting it on the pipettor, and the manufacturer's instructions were followed for further examination.

All animal experiments were performed in compliance with the NIH Guide for the Care and Use of Laboratory Animals (National Academies Press, 2011) and the ARRIVE guidelines and were approved by the Institutional Animal Care and Use Committee (IACUC) of the Shanghai Institute of Nutrition and Health, Chinese Academy of Sciences.

### 4.15 Cell Isolation Using Laser Capture Microdissection (LCM)

Tumor samples were stored at −80°C until LCM. Prior to cryosectioning, polyethylene naphthalate 1.0 (PEN) membrane slides (Zeiss) were UV‐radiated for 30 min, with tumor samples equilibrated to cryosection temperature (−27°C) and embedded into optimal cutting temperature compound (VWR International). Sections of 7–8 μm were cryosectioned onto the PEN‐membrane slides followed by cresyl violet staining. For this, the slides were immersed in 70% ethanol, followed by a dip for 10 s into 1% cresyl violet acetate solution, then dehydrated with dipping into 70% ethanol and 100% ethanol before being dried completely before LCM.

The cresyl violet stain allowed reliable histomorphologic identification of the respective sample features and manual selection of the cell populations under visual control using a 40× objective (Zeiss). The laser‐pressure catapulted microdissected cell populations were dry‐collected into sterile PALM opaque AdhesiveCap microfuge tubes (Zeiss, Germany) for cell lysis and subsequent quantitative RT‐PCR profiling.

### 4.16 Tissue SA‐β‐Gal Staining and Histological Analysis

For tissue SA‐β‐gal staining, frozen sections were dried at 37°C for 20–30 min before being fixed for 15 min at room temperature. The frozen sections were washed thrice with PBS and incubated with SA‐β‐gal staining reagent (Beyotime) overnight at 37°C. After completion of SA‐β‐gal staining, sections were stained with eosin for 1–2 min, rinsed under running water for 1 min, differentiated in 1% acid alcohol for 10–20 s, and washed again under running water for 1 min. Sections were dehydrated in increasing concentrations of alcohol and cleared in xylene. After drying, samples were examined under a brightfield microscope.

### 4.17 Masson's Trichrome Staining

Tissue collagen was stained by Masson's trichrome method (BioTnA, Kaohsiung). Paraffin sections were rehydrated through serial concentrations (100%, 95%, and 70%) of alcohol following deparaffinization. Tissue slides were stained according to the manufacturer's protocol. The slides were then dehydrated through serial concentrations (95%, 100%) of alcohol and cleared in xylene. Collagen fibers were stained blue, while muscle fibers, cellulose, and red blood cells were stained red. Fibrosis regions were quantified by ImageJ.

For each preclinical regimen, animals were monitored for conditions including hypersensitivity (changes in body temperature, altered breathing, and ruffled fur), body weight, mortality, and changes in behavior (i.e., loss of appetite and distress) and were disposed of appropriately according to the individual pathological severity as defined by relevant guidelines.

### 4.18 Biochemical Parameter Analysis

Blood samples were collected from the right retro‐orbital plexus. Creatinine (CREA), aspartate aminotransferase (AST/GOT), alanine aminotransferase (ALT/GPT), blood urea nitrogen (BUN), and triglyceride (TG) were determined in EDTA plasma by using an automated analyzer (Cobas Mira, Roche) according to the manufacturer's instructions.

### 4.19 Flow Cytometry and Analysis

Following anesthesia, mice were perfused through the heart with PBS to clear circulating blood. The liver was then excised and placed in cold PBS. For tissue dissociation, the liver was mechanically dissociated using the bottom of a syringe in 5 mL of digestion buffer (RPMI 1640 supplemented with 2% FBS and 1 mg/mL collagenase IV) in a 6‐well plate. The sample was incubated at 37°C for 1 h to ensure complete digestion. After digestion, the tissue suspension was mixed with 20 mL of HBSS containing 2% FBS and vigorously pipetted to dissociate remaining cell aggregates. The homogenate was centrifuged at 50–100 × g for 1 min to pellet hepatocytes and undigested debris. The supernatant was filtered through a 70 μm cell strainer, and the collected cells were centrifuged again. The resulting pellet was resuspended in 4 mL of HBSS with 2% FBS. For density gradient separation, the cell suspension was carefully layered over 4 mL of Ficoll‐Paque and centrifuged at 2500 rpm (approximately 400 × g) for 5 min with a low brake setting (brake 3). The non‐parenchymal cell fraction, enriched in immune cells, was collected from the intermediate layer. Finally, the isolated cells were washed once in HBSS with 2% FBS for downstream applications.

Flow cytometry was performed following standard protocols. An Fc receptor block (BioLegend) was incubated with samples to reduce nonspecific antibody binding for half an hour at 4°C. Afterwards, the cells were incubated for 0.5 h at 4°C with fluorochrome‐tagged monoclonal antibodies (BioLegend), such as anti‐mouse F4/80 antibody PE and anti‐mouse CD45 antibody. Cell populations were gated as follows: M1 macrophages (MHC‐II) and M2 macrophages (CD11B+ CD206+). We performed flow cytometry with a FACSCalibur flow cytometer (Beckman), and FlowJo software (FlowJo, LCC) was used for data analysis.

### 4.20 Preclinical Intervention of Aged Mice

Wild‐type C57BL/6J mice aged 11–12 weeks and 19 months were employed in the study for aging‐intervention purposes. To evaluate the geroprotective effects, 19 month‐old female mice were administered PQQ (5.0 mg/kg) or vehicle via intraperitoneal injection every two days over a 3‐month period. At the end of the in vivo treatment, mice were sacrificed, with liver, kidney, and spleen samples collected for analysis. Tissues were analyzed for senescence‐associated markers, including SA‐β‐gal activity and expression of hallmark SASP factors. The experimental protocol was approved by the Institutional Animal Care and Use Committee (IACUC) of the Shanghai Institute of Nutrition and Health, Chinese Academy of Sciences. All procedures were conducted strictly in accordance with IACUC guidelines for animal experiments.

### 4.21 Statistical Analysis

All in vitro experiments were performed in triplicate, while animal studies were conducted with at least 8 mice *per* group for most preclinical assays. Data are presented as mean ± SD except where otherwise indicated. GraphPad Prism 9.5.1 was used to collect and analyze data, with statistical significance determined according to individual settings. Cox proportional hazards regression model and multivariate Cox proportional hazards model analysis were performed with statistical software SPSS. Statistical significance was determined by an unpaired two‐tailed Student's *t‐*test, one‐ or two‐way ANOVA, Pearson's correlation coefficients test, Kruskal–Wallis, log‐rank test, Wilcoxon‐Mann–Whitney test, or Fisher's exact test. For all statistical tests, a *p* value < 0.05 was considered significant.

## Author Contributions

Y.S. conceived this study, designed the experiments, and orchestrated the project. B.J. performed most of the in vitro assays and in vivo experiments. H.Z. performed proteomics studies and data analysis. Q.X. provided early results from primary screening of the drug library and key suggestions for biological experiments. Z.J. performed some cell culture and drug treatment assays. Q.F. performed partial preclinical studies and provided animal data. R.H. provided critical conceptual inputs. Y.S. performed data analysis, graphic presentation, and finalized the manuscript. All authors critically read and commented on the final manuscript.

## Conflicts of Interest

The authors declare no conflicts of interest.

## Supporting information


Data S1.

## Data Availability

Data supporting the plots within this paper and other findings of this study are available from the corresponding author upon reasonable request. The RNA‐seq data generated in the present study have been deposited in the NCBI Gene Expression Omnibus (GEO) database under accession code GSE264316. The mass spectrometry proteomics data have been deposited to the ProteomeXchange Consortium via the PRIDE partner repository iProX with the dataset identifier PXD062905.
