# Supplementary material for: Pyrroloquinoline Quinone Is an Effective Senomorphic Agent to Target the Pro‐Inflammatory Phenotype of Senescent Cells
Source: Aging Cell. 2025 Jun 19;24(9):e70138. doi: 10.1111/acel.70138 (PMC12419835; doi:10.1111/acel.70138)
Supplement: Supplementary file 1 — Data S1. [file ACEL-24-e70138-s001.pdf]

Figure S1

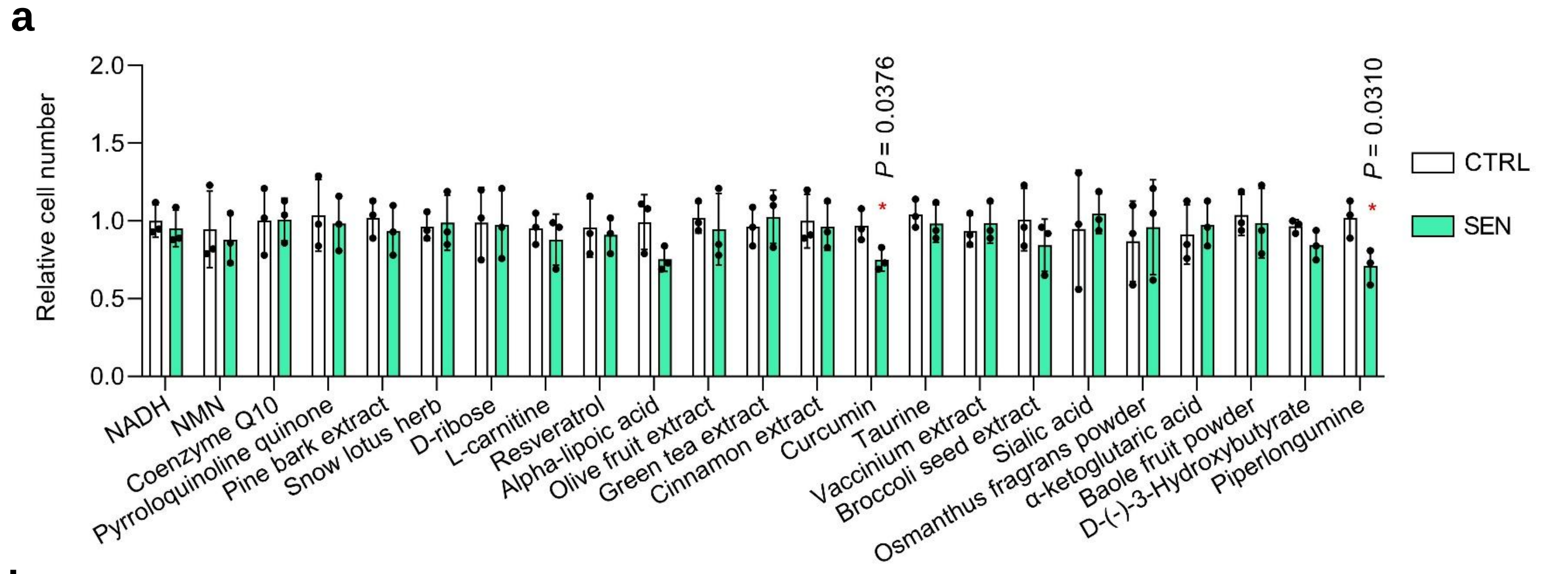

a
Relative cell number
2.0
1.5
1.0
0.5
0.0
P = 0.0376
P = 0.0310
*
*
CTRL
SEN
NADH
NMN
Coenzyme Q10
Pyrroloquinoline quinone
Pine bark extract
Snow lotus herb
D-ribose
L-carnitine
Resveratrol
Alpha-lipoic acid
Olive fruit extract
Green tea extract
Cinnamon extract
Curcumin
Taurine
Vaccinium extract
Broccoli seed extract
Sialic acid
Osmanthus fragrans powder
α-ketoglutaric acid
Baole fruit powder
D-(-)-3-Hydroxybutyrate
Piperlongumine

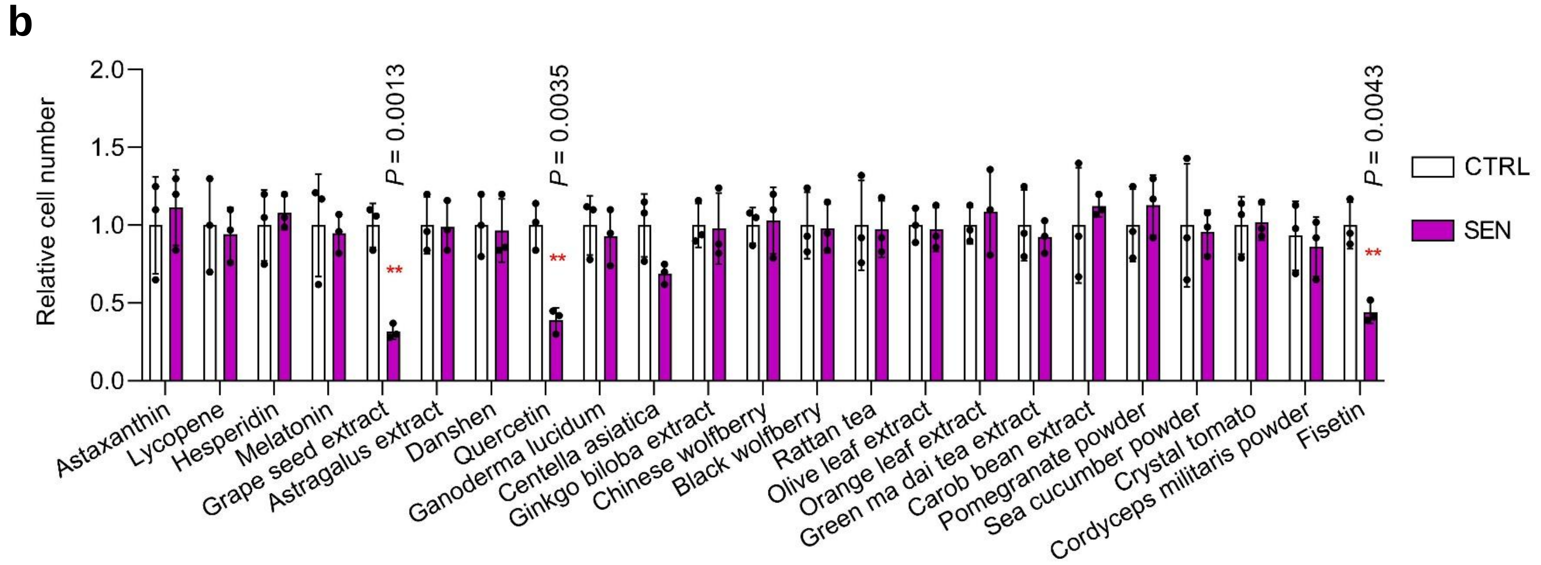

b
Relative cell number
2.0
1.5
1.0
0.5
0.0
P = 0.0013
P = 0.0035
P = 0.0043
**
**
**
CTRL
SEN
Astaxanthin
Lycopene
Hesperidin
Melatonin
Grape seed extract
Astragalus extract
Danshen
Quercetin
Ganoderma lucidum
Centella asiatica
Ginkgo biloba extract
Chinese wolfberry
Black wolfberry
Rattan tea
Olive leaf extract
Orange leaf extract
Green ma dai tea extract
Carob bean extract
Pomegranate powder
Sea cucumber powder
Crystal tomato
Cordyceps militaris powder
Fisetin

Figure S2

a

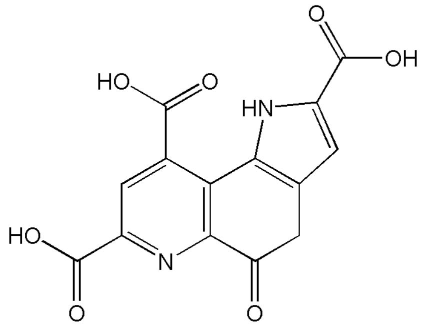

b

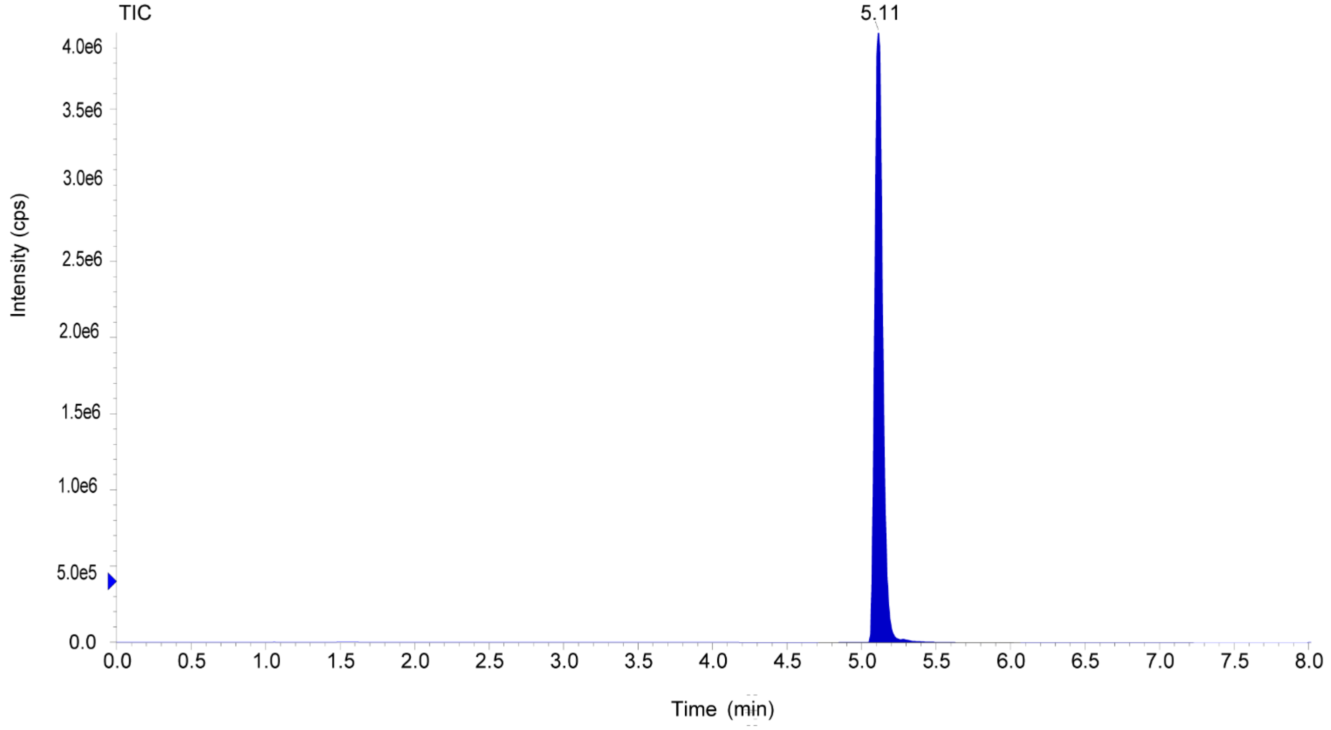

c

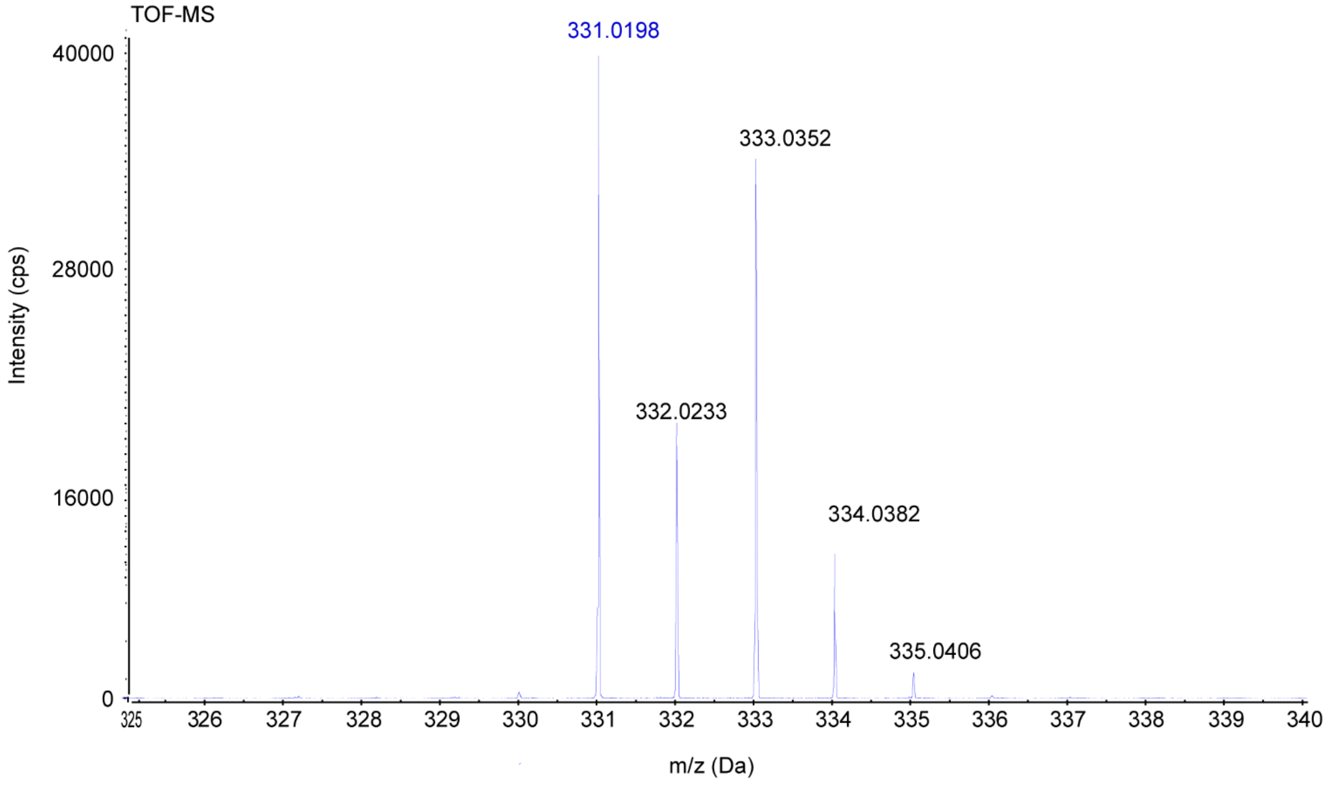

Figure S3

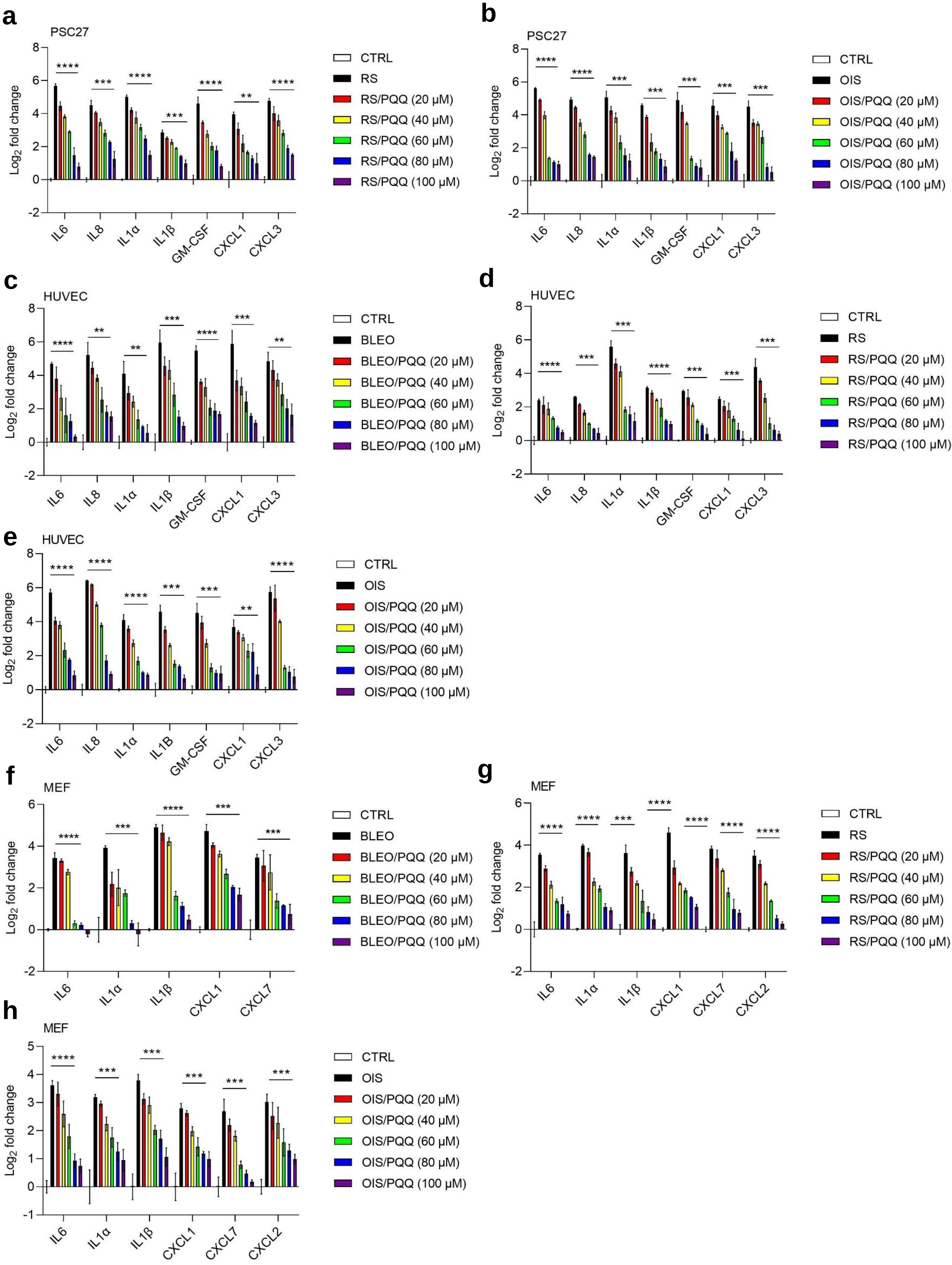
a
PSC27
Log2 fold change
IL6
IL8
IL1α
IL1β
GM-CSF
CXCL1
CXCL3
CTRL
RS
RS/PQQ (20 μM)
RS/PQQ (40 μM)
RS/PQQ (60 μM)
RS/PQQ (80 μM)
RS/PQQ (100 μM)
b
PSC27
CTRL
OIS
OIS/PQQ (20 μM)
OIS/PQQ (40 μM)
OIS/PQQ (60 μM)
OIS/PQQ (80 μM)
OIS/PQQ (100 μM)
c
HUVEC
CTRL
BLEO
BLEO/PQQ (20 μM)
BLEO/PQQ (40 μM)
BLEO/PQQ (60 μM)
BLEO/PQQ (80 μM)
BLEO/PQQ (100 μM)
d
HUVEC
CTRL
RS
RS/PQQ (20 μM)
RS/PQQ (40 μM)
RS/PQQ (60 μM)
RS/PQQ (80 μM)
RS/PQQ (100 μM)
e
HUVEC
IL1B
CTRL
OIS
OIS/PQQ (20 μM)
OIS/PQQ (40 μM)
OIS/PQQ (60 μM)
OIS/PQQ (80 μM)
OIS/PQQ (100 μM)
f
MEF
IL6
IL1α
IL1β
CXCL1
CXCL7
CTRL
BLEO
BLEO/PQQ (20 μM)
BLEO/PQQ (40 μM)
BLEO/PQQ (60 μM)
BLEO/PQQ (80 μM)
BLEO/PQQ (100 μM)
g
MEF
IL6
IL1α
IL1β
CXCL1
CXCL7
CXCL2
CTRL
RS
RS/PQQ (20 μM)
RS/PQQ (40 μM)
RS/PQQ (60 μM)
RS/PQQ (80 μM)
RS/PQQ (100 μM)
h
MEF
IL6
IL1α
IL1β
CXCL1
CXCL7
CXCL2
CTRL
OIS
OIS/PQQ (20 μM)
OIS/PQQ (40 μM)
OIS/PQQ (60 μM)
OIS/PQQ (80 μM)
OIS/PQQ (100 μM)

Figure S4

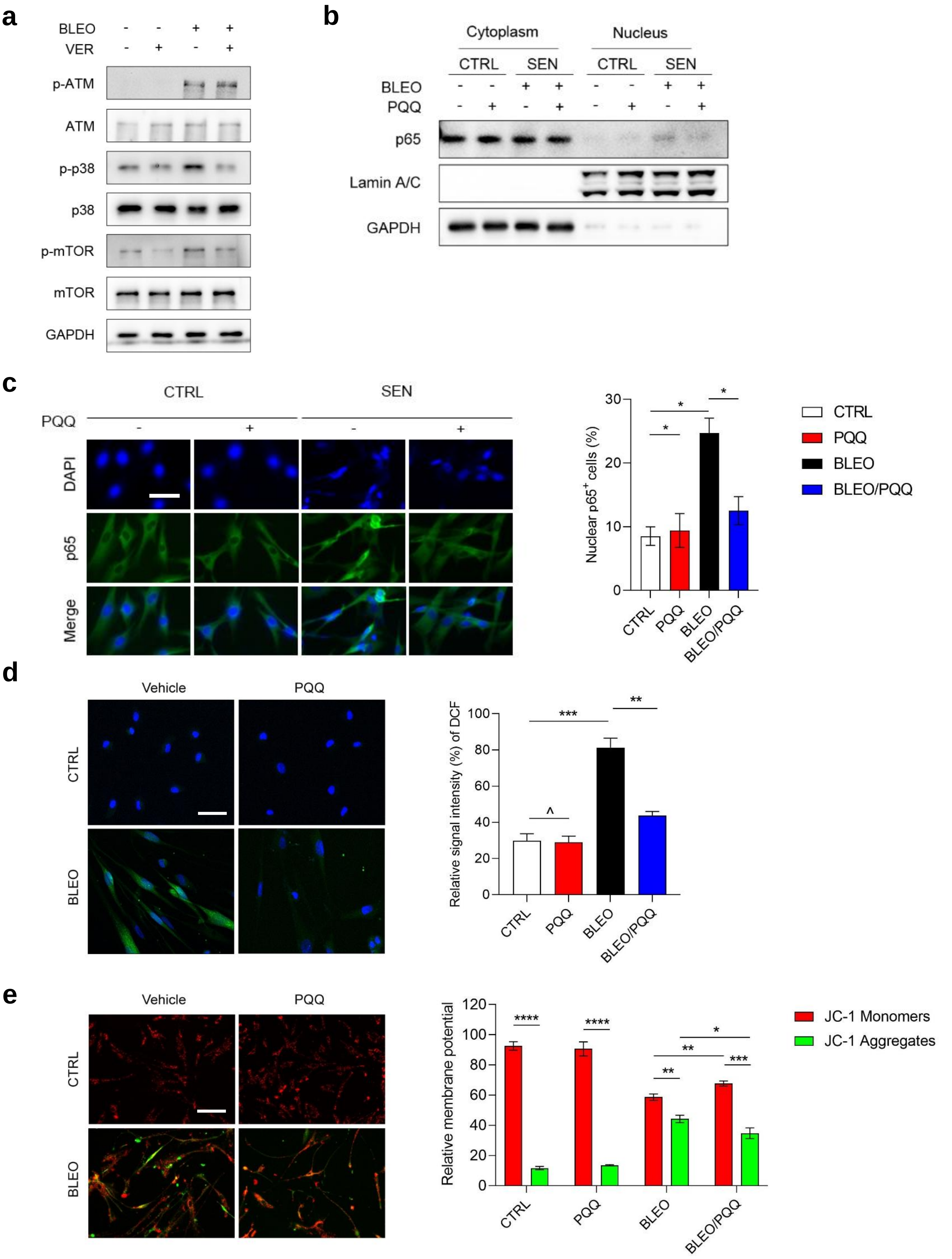
a
BLEO
VER
p-ATM
ATM
p-p38
p38
p-mTOR
mTOR
GAPDH
b
Cytoplasm
Nucleus
CTRL
SEN
BLEO
PQQ
p65
Lamin A/C
GAPDH
c
CTRL
SEN
PQQ
DAPI
p65
Merge
Nuclear p65+ cells (%)
CTRL
PQQ
BLEO
BLEO/PQQ
d
Vehicle
PQQ
CTRL
BLEO
Relative signal intensity (%) of DCF
e
Vehicle
PQQ
CTRL
BLEO
Relative membrane potential
JC-1 Monomers
JC-1 Aggregates

Figure S5

a

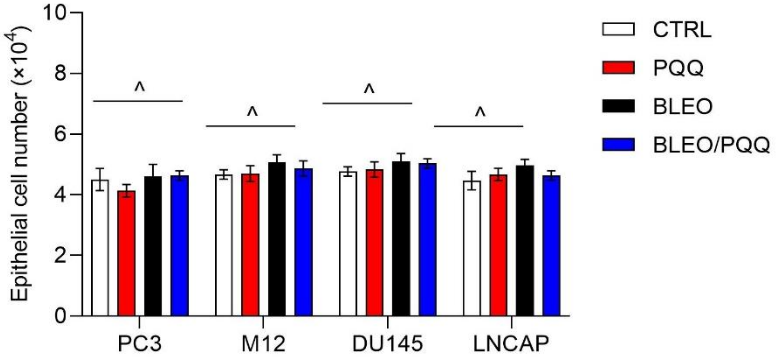
Epithelial cell number (×10^4)
10
8
6
4
2
0
PC3
M12
DU145
LNCAP
CTRL
PQQ
BLEO
BLEO/PQQ

b

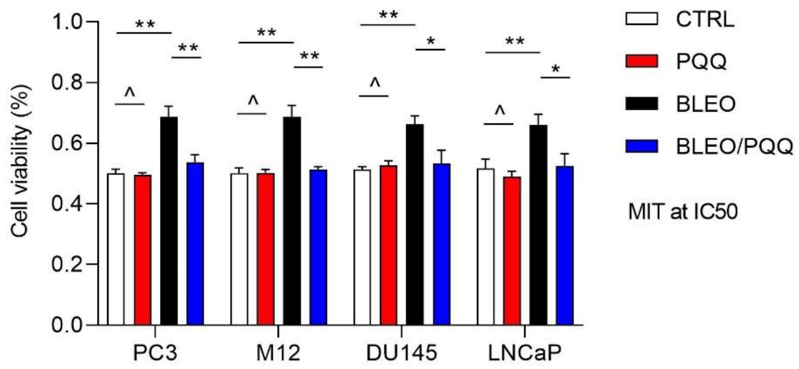
Cell viability (%)
1.0
0.8
0.6
0.4
0.2
0.0
PC3
M12
DU145
LNCaP
CTRL
PQQ
BLEO
BLEO/PQQ
MIT at IC50

Figure S6

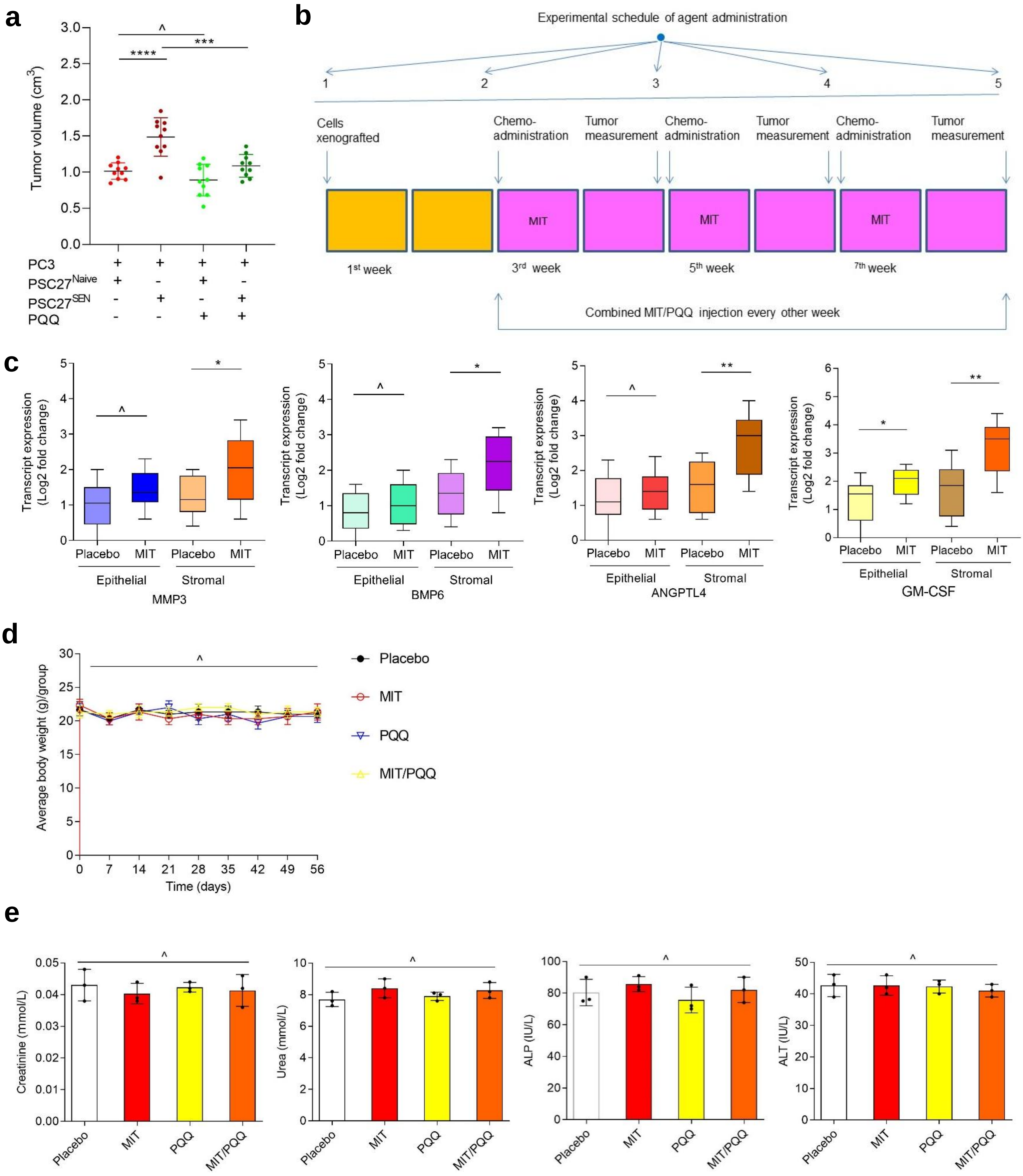
a
Tumor volume (cm3)
^
****
***
PC3
PSC27Naive
PSC27SEN
PQQ
b
Experimental schedule of agent administration
1
2
3
4
5
Cells xenografted
Chemo-administration
Tumor measurement
MIT
1st week
3rd week
5th week
7th week
Combined MIT/PQQ injection every other week
c
Transcript expression (Log2 fold change)
Placebo
MIT
Epithelial
Stromal
MMP3
BMP6
ANGPTL4
GM-CSF
d
Average body weight (g)/group
Time (days)
Placebo
MIT
PQQ
MIT/PQQ
e
Creatinine (mmol/L)
Urea (mmol/L)
ALP (IU/L)
ALT (IU/L)

Figure S7

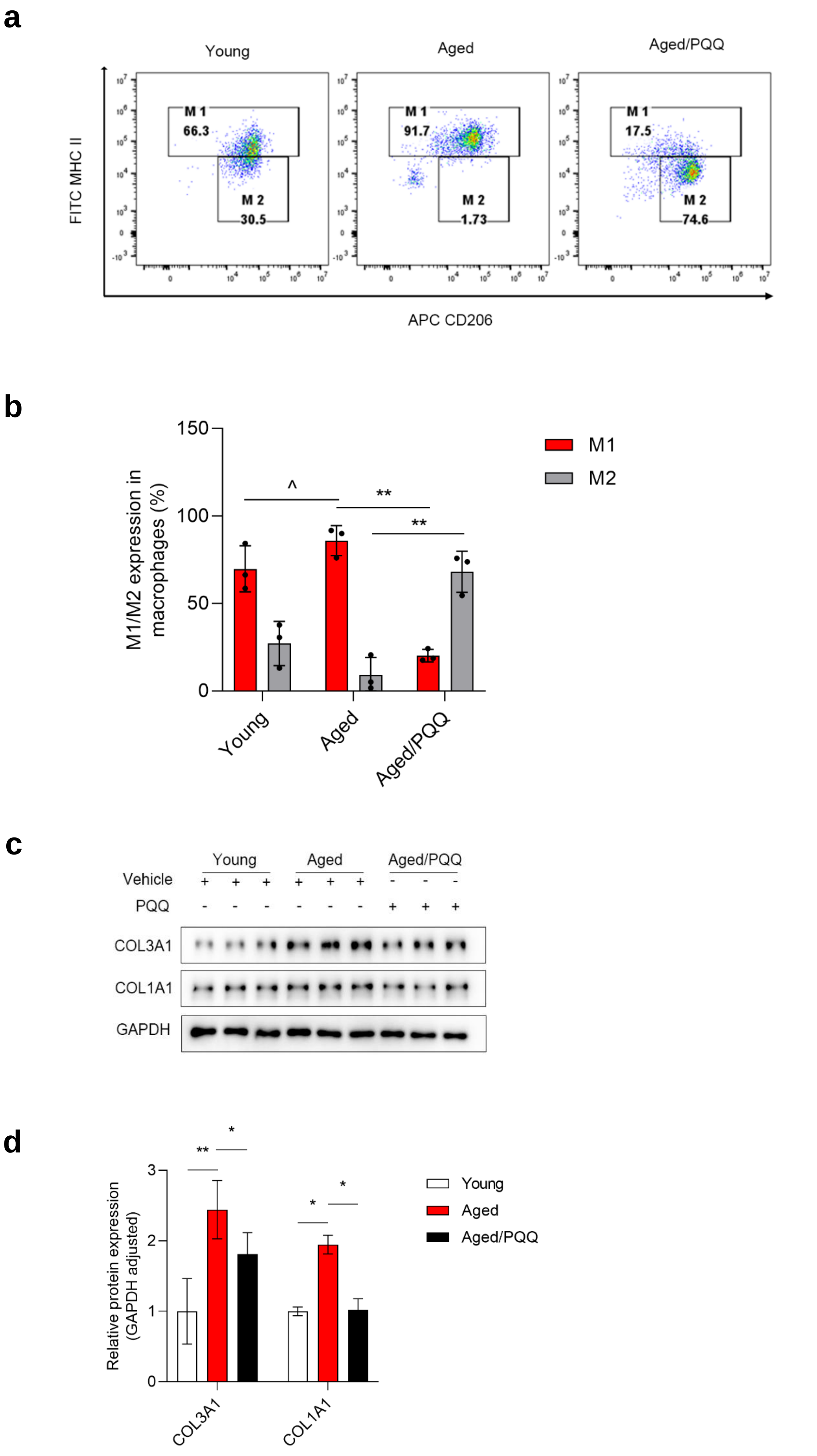

a
Young
Aged
Aged/PQQ
FITC MHC II
APC CD206
M 1
66.3
M 2
30.5
M 1
91.7
M 2
1.73
M 1
17.5
M 2
74.6
b
M1/M2 expression in macrophages (%)
150
100
50
0
^
**
**
Young
Aged
Aged/PQQ
M1
M2
c
Young
Aged
Aged/PQQ
Vehicle + + + + + + - - -
PQQ - - - - - - + + +
COL3A1
COL1A1
GAPDH
d
Relative protein expression (GAPDH adjusted)
3
2
1
0
**
*
*
*
COL3A1
COL1A1
Young
Aged
Aged/PQQ

Figure S8

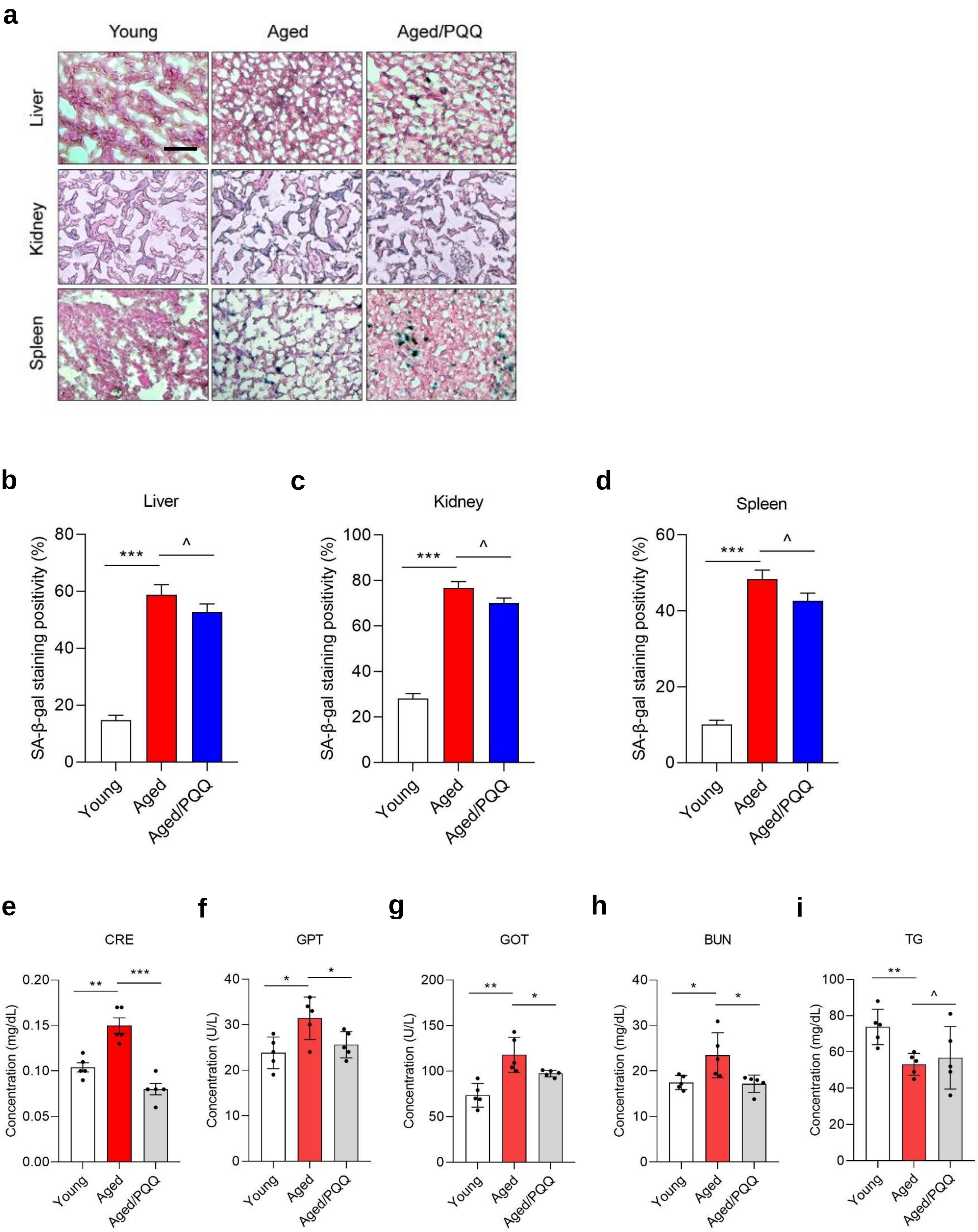
a
Young
Aged
Aged/PQQ
Liver
Kidney
Spleen
b
Liver
c
Kidney
d
Spleen
SA-β-gal staining positivity (%)
***
^
Young
Aged
Aged/PQQ
e
CRE
f
GPT
g
GOT
h
BUN
i
TG
Concentration (mg/dL)
Concentration (U/L)
**
***
*
^

Figure S9

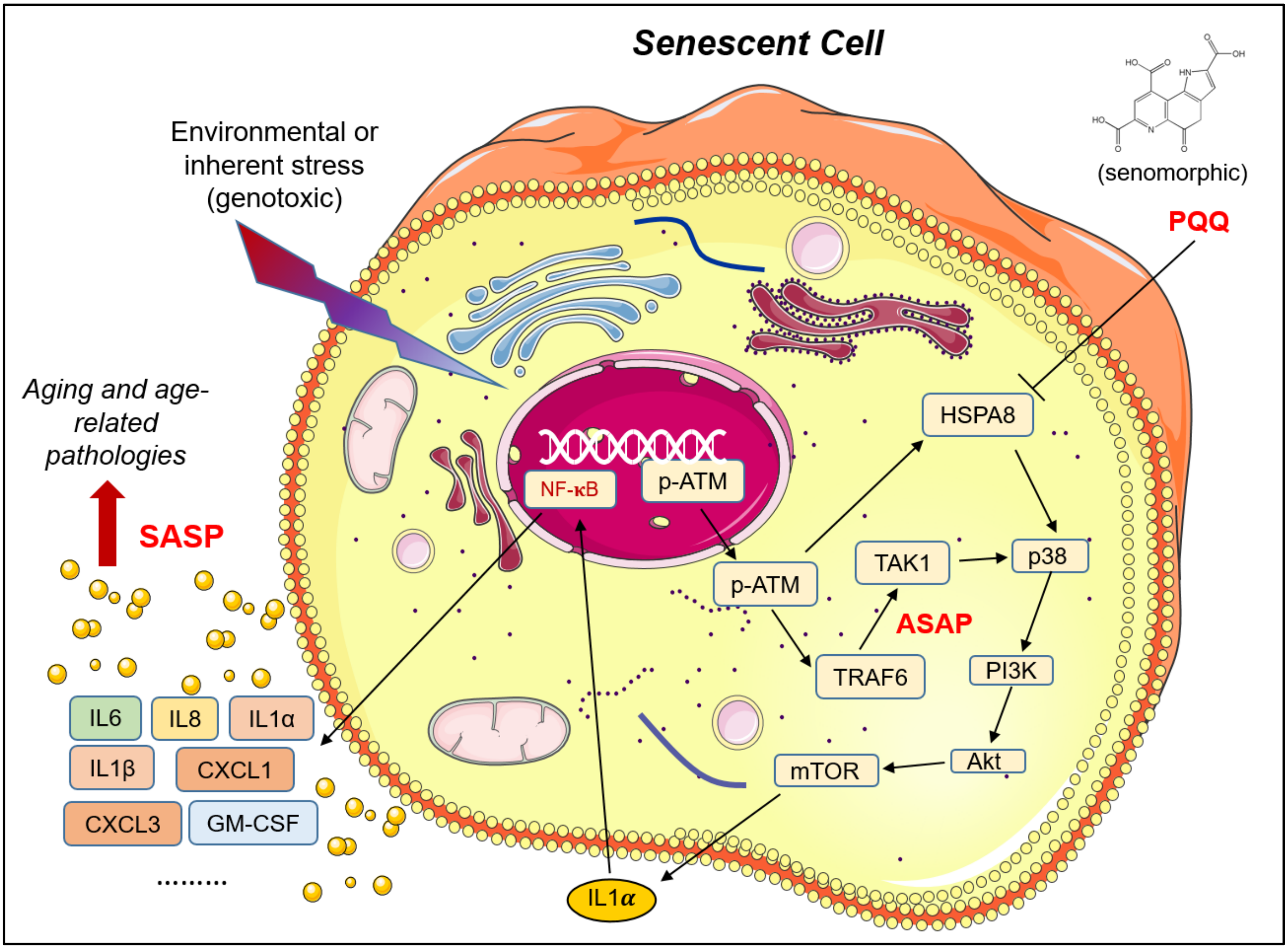

Senescent Cell
Environmental or inherent stress (genotoxic)
(senomorphic)
PQQ
Aging and age-related pathologies
SASP
NF-κB
p-ATM
p-ATM
HSPA8
TAK1
p38
ASAP
TRAF6
PI3K
Akt
mTOR
IL1α
IL6
IL8
IL1α
IL1β
CXCL1
CXCL3
GM-CSF
....

Supplementary Figure Legends

Figure S1. Assessment of *in vitro* effects of individual agents on the survival of PSC27 cells in culture.

(**a**) Evaluation of the effects of individual agents of group A on the survival of cells in culture. (**b**) Appraisal of the effects of individual agents of group B on the survival of cells in culture. Piperlongumine and fisetin, positive senolytic controls. CTRL, control. SEN, senescent. In each graph, red stars indicate statistical significance. Data in **a**-**b** are shown as mean ± SD and representative of 3 independent biological replicates. *, $P < 0.05$; **, $P < 0.01$.

Figure S2. Chemical structure and high resolution mass spectra of PQQ.

(**a**) Chemical structure of p*yrroloquinoline quinone (*PQQ). (**b,c**) High resolution mass spectra showing the total ion chromatogram (TIC, **b**) and base peak chromatogram (BPC, **c**) of a chemically pure sample PQQ after performance of HPLC-ESI-QTOF-MS. cps, counts *per* second.

Figure S3. Measurement of the effect of PQQ on SASP expression in different cell lines.

(**a**-**b**) Quantitative transcriptional analysis of typical SASP factor expression in PSC27 cells upon RS (**a**) or OIS (**b**) in the absence or presence of PQQ applied at increasing concentrations (20-100 μM). (**c**-**e**) Quantitative transcriptional analysis of typical SASP factor expression in HUVEC cells upon BLEO-induced senescence (**c**), RS (**d**) or OIS (**e**) in the absence or presence of PQQ applied at increasing concentrations (20-100 μM). (**f**-**h**) Quantitative transcriptional analysis of typical SASP factor expression in MEF cells upon BLEO-induced senescence (**f**), RS (**g**) or OIS (**h**) in the absence or presence of PQQ applied at increasing concentrations (20-100 μM). RS, replicative senescence. OIS, oncogene-induced senescence.

HUVEC, human umbilical vein endothelial cell. MEF, mouse embryonic fibroblast. Data in **a**-**h** are shown as mean ± SD and representative of 3 independent biological replicates. *P* values calculated by two-sided *t*-tests. ^, $P > 0.05$; *, $P < 0.05$; **, $P < 0.01$; ***, $P < 0.001$; ****, $P < 0.0001$.

Figure S4. Immunoblot assays to examine the influence of HSPA8 inhibitor and PQQ on signaling activities, as well as PQQ on ROS production and mitochondrial membrane potential of senescent cells.

(**a**) Immunoblot analysis of the effect of VER on DNA damage response and downstream pathways. VER, VER155008, a small molecule inhibitor of HSPA8. GAPDH, loading control. (**b**) Immunoblot examination of p65 (RelA) distribution between cytoplasm and nuclei of cells. Lamin A/C and GAPDH, loading controls of nuclear and cytoplasmic proteins, respectively. (**c**) Immunofluorescence analysis of p65 nuclear translocation upon treatment by PQQ in CTRL or SEN cells. Right, statistics. (**d**) Measurement of ROS with 2′-7′- dichlorodihydrofluorescein diacetate (DCFH-DA), a cell-permeable fluorescent probe sensitive to cellular redox state changes. Experiments were performed 1 d after PQQ treatment. Left, representative images. Scale bar, 10 μm. Right, comparative statistics. ROS, reactive oxygen species. (**e**) Assessment of the mitochondrial membrane potential (Δψm) with JC-1 assay. Green fluorescence, JC-1 monomers. Red, JC-1 aggregates. PQQ was used at 100 μM in these assays. Scale bar, 15 μm. Data in **c**-**e** are shown as mean ± SD and representative of 3 independent biological replicates. *P* values were calculated by two-sided *t*-tests. ^, $P > 0.05$; *, $P < 0.05$; **, $P < 0.01$; ***, $P < 0.001$; ****, $P < 0.0001$.

Figure S5. Measurement of proliferation and chemoresistance of PCa cell lines in remarkably shortened periods.

(**a**-**b**) PCa cell lines (PC3, M12, DU145 and LNCaP) were cultured for 24 h with CM from PSC27 stromal sublines, and subject to proliferation assays (**a**) and

chemoresistance (**b**) tests. Data are representative of 3 independent experiments. All P values were calculated by two-sided t-tests. ^, $P > 0.05$; *, $P < 0.05$; **, $P < 0.01$.

Figure S6. Senescent stromal cells confer therapeutic resistance on solid tumors but can be reversed by PQQ in preclinical conditions.

(**a**) Statistical measurements of tumor volume at the end of an 8-week growth period. PC3 cells were xenografted either alone or together with PSC27 cells to the hind flank of SCID animals. Before subcutaneous implantation, PSC27 cells were naïve or subject to senescence induction by BLEO ($PSC27^{Naive}$ and $PSC27^{SEN}$, respectively). (**b**) A strategic diagram of drug administration and tumor surveillance in the preclinical trial. PC3 cells alone or combined with PSC27 cells were inoculated subcutaneously to SCID mice 2 weeks prior to chemotherapy. MIT was provided on the first day of each week starting from the 3rd week, then given every other week with a total number of 3 doses. As a candidate of senomorphics, PQQ was given 12 h before each time of MIT delivery (a total of 3 doses throughout the regimen). At the end of 8 weeks, mice were sacrificed, with tumor volume measured before subject to histological assays. (**c**) Quantitative expression analysis at transcript level of a subset of core SASP factors expressed in stromal cells isolated from tumors of SCID mice. Tumor tissues from animals implanted with both stromal and cancer cells in xenografts were subject to laser capture microdissection (LCM), cell lineage isolation, RNA preparation and qRT-PCR analyses. (**d**) Mouse body weight determination performed once a week until the end of the therapeutic regimen. MIT (0.2 mg/kg) was administered either alone or with the senomorphic agent PQQ (5.0 mg/kg) on 1st day of week 3, 5 and 7 after tumor implantation (PC3/PSC27) to SCID animals. (**e**) Terminal bleeds were taken *via* cardiac punctures on day 56. Creatinine, urea, alkaline phosphatase (ALP) and alanine aminotransferase (ALT) at the serum level were analyzed for toxicity

appraisal with SCID mice developing prostate tumors. Data are shown as mean ± SD and representative of 3 independent experiments. N = 3 *per* treatment arm. MIT, mitoxantrone. *P* values were calculated by Student's *t*-tests (**a**, **c**, **d**) or one-way ANOVA (**e**). ^, $P > 0.05$. *$P < 0.05$. **$P < 0.01$. ***$P < 0.001$. ****$P < 0.0001$.

Figure S7. Evaluation of macrophage phenotypic shift and collagen expression change in solid organs of aged mice.

(**a**) Flow cytometry profiling of phenotypic shift between M1 and M2 in the liver of mice. Sorting analysis with anti-F4/80 and anti-CD45 was performed, gated with channel FITC MHC-II and channel APC CD206, respectively. (**b**) Quantification of M1 and M2 macrophages as described in (**a**). (**c**) Immunoblot analysis of COL3A1 and COL1A1 expression in mouse tissues isolated from kidneys to assess potential development of renal fibrosis. GAPDH, loading control. (**d**) Relative expression of COL3A1 and COL1A1 was determined with samples described in (**c**). *P* values were calculated by Student's *t*-tests (b, d). ^, $P > 0.05$. *$P < 0.05$. **$P < 0.01$. ***$P < 0.001$. ****$P < 0.0001$.

Figure S8. Assessment of tissue level senescence in solid organs of aging mice.

(**a**) A combined H&E and SA-β-gal staining was performed on tissue sections from liver, kidney and spleen to determine tissue level senescence. Mice were either young, aged or aged subject to PQQ intervention. (**b**-**d**) Comparative statistics of SA-β-gal staining positivity in tissue sections of liver, kidney and spleen. N = 3 *per* treatment arm. Data are shown as mean ± SD and representative of 3 independent experiments (**b**-**d)**. (**e**-**i**) Measurement of plasma levels of creatinine (CRE)(**e**), aspartate aminotransferase (GPT)(**f**), alanine aminotransferase (GOT)(**g**), blood urea nitrogen (BUN)(**h**) and triglyceride (TG)(**i**). *P* values were calculated by Student's *t*-tests. ^, $P > 0.05$. *$P < 0.05$. **$P < 0.01$. ***$P < 0.001$.

Figure S9. Mechanistic working model of PQQ in acting as a novel senomorphic agent by restraining the SASP of senescent cells.

Intracellular signaling of cellular senescence usually starts from DNA damage response, which can be caused by environmental or inherent stresses. During the early stage or acute stress associated-phenotype (ASAP) phase, ATM is phosphorylated and translocated from nuclei to the cytoplasm. HSPA8 mediates signal transduction from ATM to p38, although the TRAF6/TAK1/p38 pathway is activated in parallel to allow the culmination of signaling activities towards a full spectrum of SASP. As a senomorphic agent, PQQ physically interacts with one of its direct targets HSPA8, resulting in abrogated signal transduction, activation of downstream factors and significantly reduced expression of the SASP. PQQ holds prominent potential to delay chronological aging and ameliorate age-related conditions by acting as an effective senomorphic agent.

Table S1. A full list of candidate agents of the natural product library screened in this study.

No.	***Agent name***
1	NADH
2	NMN
3	Coenzyme Q10
4	Pyrroloquinoline quinone
5	Pine bark extract
6	Snow lotus herb
7	D-ribose
8	L-carnitine
9	Resveratrol
10	Alpha-lipoic acid (thiotic acid)
11	Olive fruit extract
12	Green tea extract
13	Cinnamon extract
14	Curcumin
15	Astaxanthin
16	Astragalus extract
17	Hesperidin
18	Grape seed extract
19	Lycopene
20	Melatonin
21	Danshen (Salvianolic acid A)
22	Quercetin
23	Ganoderma lucidum
24	Ginkgo biloba extract
25	Chinese wolfberry
26	Centella asiatica
27	Taurine
28	Vaccinium extract
29	Broccoli seed extract
30	Sialic acid

31	Osmanthus fragrans powder
32	Cordyceps militaris powder
33	Black wolfberry frozen dried powder
34	Rattan tea instant powder
35	Olive leaf extract
36	Orange olive leaf extract
37	Green ma dai tea extract
38	Carob bean extract
39	Pomegranate powder extract
40	Sea cucumber powder
41	Crystal tomato
42	α-ketoglutaric acid
43	Fisetin
44	Baole fruit powder
45	D-(-)-3-Hydroxybutyrate sodium
46	Piperlongumine

Table S2. Data from drug affinity responsive target stability and LC-MS/MS proteomics

Protein FDR Confidence: Combined	Accession	Gene Symbol	Description	Exp. q-value: Combined	Sum PEP Score	Coverage [%]	# Peptides	# PSMs	# Unique Peptides	# AAs	MW [kDa]	calc. pI	durg_VS_con_Ratio	durg_VS_con_P value	Mean_durg	Mean_con	Abundance: durg1	Abundance: durg2	Abundance: durg3	Abundance: con1	Abundance: con2	Abundance: con3
High	Q00610	CLTC	Clathrin heavy chain 1 OS=Homo sapiens OX=9606 GN=CLTC PE=1 SV=5	0	1293.612	58	80	595	60	1675	191.5	5.69	0.815051771	0.047891522	1307411102	1604083505	1479944397	1231997687	1210291222	1723224771	1532506921	1556518824
High	P46940	IQGAP1	Ras GTPase-activating-like protein IQGAP1 OS=Homo sapiens OX=9606 GN=IQGAP1 PE=1 SV=1	0	821.337	47	65	338	63	1657	189.1	6.48	0.792227726	0.041093241	621306733.5	784252700.3	701605581	583524726.9	578789892.5	858865917.7	749326502.9	744565680.4
High	P60709	ACTB	Actin, cytoplasmic 1 OS=Homo sapiens OX=9606 GN=ACTB PE=1 SV=1	0	778.934	83	29	1017	14	375	41.7	5.48	0.824280715	0.04618781	1968824253	2388536110	2225546747	1904893517	1776032494	2497608797	2382545797	2285453735
High	P07814	EPRS1	Bifunctional glutamate/proline--tRNA ligase OS=Homo sapiens OX=9606 GN=EPRS1 PE=1 SV=5	0	576.623	43	48	217	48	1512	170.5	7.33	0.806336183	0.046995794	294988947.7	365838658.7	338052102.3	272524993.3	274389747.7	387696806.9	365890967	343928202.4
High	P11142	**HSPA8**	Heat shock cognate 71 kDa protein OS=Homo sapiens OX=9606 GN=HSPA8 PE=1 SV=1	0	562.157	66	34	391	24	646	70.9	5.52	0.710451744	0.042649526	1316764941	1853419254	1581891469	1073005590	1295397764	2008057016	1907852162	1644348584
High	P26038	MSN	Moesin OS=Homo sapiens OX=9606 GN=MSN PE=1 SV=3	0	497.256	63	38	255	25	577	67.8	6.4	0.765662815	0.042783825	957667853.3	1250769704	1058583424	847572764.9	966847371	1405423912	1202996932	1143888270
High	Q99798	ACO2	Aconitate hydratase, mitochondrial OS=Homo sapiens OX=9606 GN=ACO2 PE=1 SV=2	0	496.957	57	35	171	35	780	85.4	7.61	0.780585707	0.042834051	425010486.1	544476387.7	490820758.4	419866340.4	364344359.5	569301099	509527535.7	554600528.4
High	Q13200	PSMD2	26S proteasome non-ATPase regulatory subunit 2 OS=Homo sapiens OX=9606 GN=PSMD2 PE=1 SV=3	0	471.052	49	33	197	33	908	100.1	5.2	0.78053218	0.033915982	240196611.8	307734412.4	268215482	230393933.4	221980420.1	335491565.6	280478479.9	307233191.7
High	Q71U36	TUBA1A	Tubulin alpha-1A chain OS=Homo sapiens OX=9606 GN=TUBA1A PE=1 SV=1	0	442.14	69	24	331	1	451	50.1	5.06	0.514121713	0.002948891	15311336.92	29781541.08	14725886.25	18352717.5	12855407	31675720.5	30962167.5	26706735.25
High	P20591	MX1	Interferon-induced GTP-binding protein Mx1 OS=Homo sapiens OX=9606 GN=MX1 PE=1 SV=4	0	396.675	52	29	214	29	662	75.5	5.83	0.786862664	0.047749167	458384827.3	582547435.9	541238470.7	395207998	438708013.2	578236199.5	571531655.9	597874452.2
High	Q9BUF5	TUBB6	Tubulin beta-6 chain OS=Homo sapiens OX=9606 GN=TUBB6 PE=1 SV=1	0	349.525	64	20	224	10	446	49.8	4.88	0.66010701	0.00870509	85155266.71	129002215.4	93484501.09	92055570.33	69925728.72	118922802.7	134824115.2	133259728.4
High	P04843	RPN1	Dolichyl-diphosphooligosaccharide--protein glycosyltransferase subunit 1 OS=Homo sapiens OX=9606 GN=RPN1 PE=1 SV=1	0	334.952	48	21	130	21	607	68.5	6.38	0.795442445	0.036168306	246647949.8	310076425.1	279319009.4	224314042.6	236310797.3	332980686.3	293651228.7	303597360.4
High	P60842	EIF4A1	Eukaryotic initiation factor 4A-I OS=Homo sapiens OX=9606 GN=EIF4A1 PE=1 SV=1	0	331.534	60	22	189	11	406	46.1	5.48	0.744157901	0.010108039	247808390.8	333005119.8	273407207.8	228423927.7	241594037	358696527.6	318217193.5	322101638.4
High	Q13509	TUBB3	Tubulin beta-3 chain OS=Homo sapiens OX=9606 GN=TUBB3 PE=1 SV=2	0	301.889	58	19	261	5	450	50.4	4.93	0.698368037	0.011292479	24199160.48	34651013.79	28014553.75	21985806.02	22597121.69	37143830.88	32447089.47	34362121.03
High	P50990	CCT8	T-complex protein 1 subunit theta OS=Homo sapiens OX=9606 GN=CCT8 PE=1 SV=4	0	300.717	57	27	137	27	548	59.6	5.6	0.742731377	0.034704186	228094765.3	307102638.3	263909301.6	216287088.7	204087905.7	341634308.6	289049138.2	290624468.1
High	P00367	GLUD1	Glutamate dehydrogenase 1, mitochondrial OS=Homo sapiens OX=9606 GN=GLUD1 PE=1 SV=2	0	297.081	49	24	118	24	558	61.4	7.8	0.798237158	0.040618379	222357555.5	278560767.8	255629273.7	211726700.6	199716692.1	290032340.8	262826993.1	282822969.4
High	P05556	ITGB1	Integrin beta-1 OS=Homo sapiens OX=9606 GN=ITGB1 PE=1 SV=2	0	294.948	31	23	155	23	798	88.4	5.39	0.8216902	0.046727866	485608104.5	590986852	558840128.3	456125125.4	441859059.9	591195316.5	583903443.1	597861796.5
High	P05023	ATP1A1	Sodium/potassium-transporting ATPase subunit alpha-1 OS=Homo sapiens OX=9606 GN=ATP1A1 PE=1 SV=1	0	288.028	39	33	127	23	1023	112.8	5.49	0.796526858	0.044210846	112022670.9	140638912.3	123895804.9	99483906.28	112688301.6	154258836.3	131806215.5	135851685.1
High	Q08211	DHX9	ATP-dependent RNA helicase A OS=Homo sapiens OX=9606 GN=DHX9 PE=1 SV=4	0	281.912	27	27	108	27	1270	140.9	6.84	0.686664425	0.003684879	92185061.54	134250527.9	102870485.6	93941477.29	79743221.75	132224841.6	133211656.6	137315085.3
High	P19367	HK1	Hexokinase-1 OS=Homo sapiens OX=9606 GN=HK1 PE=1 SV=3	0	281.697	42	34	147	30	917	102.4	6.8	0.799371339	0.028481429	225534162.2	282139415.3	247183237	223779360.9	205639888.7	302403639.3	261372482.6	282642124
High	P08670	VIM	Vimentin OS=Homo sapiens OX=9606 GN=VIM PE=1 SV=4	0	258.232	58	27	153	22	466	53.6	5.12	0.716028027	0.004739298	547479580.9	764606356.1	607501892.7	503900347.5	531036502.6	806060796.9	729473090.9	758285180.5
High	P54652	HSPA2	Heat shock-related 70 kDa protein 2 OS=Homo sapiens OX=9606 GN=HSPA2 PE=1 SV=1	0	252.421	30	19	131	9	639	70	5.74	0.696537495	0.014831497	21133852.11	30341298.58	24972352.75	18492339.06	19936864.53	29451278.38	32503962.88	29068654.48
High	P12268	IMPDH2	Inosine-5'-monophosphate dehydrogenase 2 OS=Homo sapiens OX=9606 GN=IMPDH2 PE=1 SV=2	0	247.99	37	14	76	13	514	55.8	6.9	0.738100305	0.022823515	62266723.1	84360787.63	68448238.5	57176720.3	61175210.5	91916955.13	74440834.46	86724573.31
High	P62942	FKBP1A	Peptidyl-prolyl cis-trans isomerase FKBP1A OS=Homo sapiens OX=9606 GN=FKBP1A PE=1 SV=2	0	247.706	59	6	89	6	108	11.9	8.16	0.763294375	0.04663224	149920871.6	196412912.9	166939249.9	150361887.1	132461477.9	221533371.9	178324432.9	189380933.9
High	P31948	STIP1	Stress-induced-phosphoprotein 1 OS=Homo sapiens OX=9606 GN=STIP1 PE=1 SV=1	0	247.676	45	27	115	27	543	62.6	6.8	0.671047694	0.021382238	207450632.8	309144394.2	243940976.1	190978870.8	187432051.5	350774959.8	289432904.8	287225318.2
High	P15311	EZR	Ezrin OS=Homo sapiens OX=9606 GN=EZR PE=1 SV=4	0	233.269	39	27	146	14	586	69.4	6.27	0.742966625	0.043366671	151503144.8	203916487.8	177560918	127407456.6	149541059.8	223750206.9	187541436.1	200457820.3
High	P50454	SERPINH1	Serpin H1 OS=Homo sapiens OX=9606 GN=SERPINH1 PE=1 SV=2	0	227.874	41	16	105	16	418	46.4	8.69	0.732001918	0.020967516	263605303	360115590.8	305310970.8	235330353.9	250174584.5	390434737.5	345158473.4	344753561.6
High	P50991	CCT4	T-complex protein 1 subunit delta OS=Homo sapiens OX=9606 GN=CCT4 PE=1 SV=4	0	227.598	43	18	112	17	539	57.9	7.83	0.788288663	0.033658207	159131743.6	201869887.4	180507872.3	157342266.8	139545091.6	210512716.3	189469645.3	205627300.7
High	Q14203	DCTN1	Dynactin subunit 1 OS=Homo sapiens OX=9606 GN=DCTN1 PE=1 SV=3	0	212.586	26	25	73	25	1278	141.6	5.81	0.73239535	0.002551542	52502086.09	71685444.35	56694820.69	52704028.59	48107409	73095830.63	68863886.81	73096615.63
High	Q14240	EIF4A2	Eukaryotic initiation factor 4A-II OS=Homo sapiens OX=9606 GN=EIF4A2 PE=1 SV=2	0	211.503	47	18	106	7	407	46.4	5.48	0.789453559	0.007379858	31474412.4	39868605.38	34130941.63	31688046.81	28604248.75	40496997.75	38907680.78	40201137.59
High	P22570	FDXR	NADPH:adrenodoxin oxidoreductase, mitochondrial OS=Homo sapiens OX=9606 GN=FDXR PE=1 SV=3	0	211.262	52	20	103	20	491	53.8	8.44	0.74170847	0.033754928	130466508.5	175899984.6	146300572.8	122813493.5	122285459.3	198573058.6	170994188	158132707.3
High	P61160	ACTR2	Actin-related protein 2 OS=Homo sapiens OX=9606 GN=ACTR2 PE=1 SV=1	0	195.844	38	14	110	14	394	44.7	6.74	0.789859019	0.024395064	269200467.9	340820907.7	294618126.8	269261258.4	243722018.4	360443409.8	313579239.3	348440073.9
High	Q14192	FHL2	Four and a half LIM domains protein 2 OS=Homo sapiens OX=9606 GN=FHL2 PE=1 SV=3	0	193.917	71	16	93	16	279	32.2	7.55	0.639297046	0.003854316	118802539.6	185833080.7	132300701.5	107353459.8	116753457.7	201726361.6	172911547	182861333.6
High	P36776	LONP1	Lon protease homolog, mitochondrial OS=Homo sapiens OX=9606 GN=LONP1 PE=1 SV=2	0	191.208	28	20	65	20	959	106.4	6.39	0.679153045	0.026549737	55620126.33	81896307.07	63646958.88	49655205.6	53558214.5	94693519.81	74420287.16	76575114.25
High	O43242	PSMD3	26S proteasome non-ATPase regulatory subunit 3 OS=Homo sapiens OX=9606 GN=PSMD3 PE=1 SV=2	0	190.54	43	21	79	21	534	60.9	8.44	0.784509368	0.011644424	89396817.31	113952517.2	97586357.31	89217203	81386891.63	118636518.8	108273724.5	114947308.4
High	P27105	STOM	Stomatin OS=Homo sapiens OX=9606 GN=STOM PE=1 SV=3	0	189.143	57	13	79	13	288	31.7	7.88	0.712281208	0.00476206	259755353.4	364680901.8	285366207.7	235345097.8	258554754.8	387744043.7	353918061.9	352380599.8
High	P31946	YWHAB	14-3-3 protein beta/alpha OS=Homo sapiens OX=9606 GN=YWHAB PE=1 SV=3	0	188.157	66	14	170	6	246	28.1	4.83	0.66005283	0.006812308	184539294.6	279582612.4	201939609.5	189102711	162575563.4	251004207.6	290410449	297333180.6
High	P12109	COL6A1	Collagen alpha-1(VI) chain OS=Homo sapiens OX=9606 GN=COL6A1 PE=1 SV=3	0	185.996	23	14	54	14	1028	108.5	5.43	0.729549612	0.011253587	31084012.13	42607125.82	36096349.25	28439834.13	28715853	43875616.25	41739156.91	42206604.31
High	P54577	YARS1	Tyrosine--tRNA ligase, cytoplasmic OS=Homo sapiens OX=9606 GN=YARS1 PE=1 SV=4	0	184.08	45	25	113	25	528	59.1	7.05	0.780570548	0.044743825	200870440.3	257337970.1	231310640.7	188084413.9	183216266.2	281525321.2	242267182.5	248221406.7
High	Q9Y4L1	HYOU1	Hypoxia up-regulated protein 1 OS=Homo sapiens OX=9606 GN=HYOU1 PE=1 SV=1	0	182.701	22	16	62	16	999	111.3	5.22	0.707060215	0.024786395	73747810.99	104302023.2	82281431.38	68388295.55	70573706.06	119090341.4	99764215.81	94051512.5
High	P35527	KRT9	Keratin, type I cytoskeletal 9 OS=Homo sapiens OX=9606 GN=KRT9 PE=1 SV=3	0	181.878	43	16	58	16	623	62	5.24	1.558542611	0.013056265	68675058.42	44063638.64	76512687.56	72061652.64	57450835.06	44330943.78	44736646.44	43123325.69
High	P27348	YWHAQ	14-3-3 protein theta OS=Homo sapiens OX=9606 GN=YWHAQ PE=1 SV=1	0	176.862	52	14	155	9	245	27.7	4.78	0.65930471	0.002253222	99938194.58	151581193.1	97464037.25	112426134.5	89924412	156165821.8	153620466.9	144957290.8
High	P12955	PEPD	Xaa-Pro dipeptidase OS=Homo sapiens OX=9606 GN=PEPD PE=1 SV=3	0	176.195	36	14	50	14	493	54.5	6	0.705852531	0.020440204	57290176.19	81164511.95	64802986.31	49461816.81	57605725.44	90058958.63	74438678.36	78995898.88
High	P10809	HSPD1	60 kDa heat shock protein, mitochondrial OS=Homo sapiens OX=9606 GN=HSPD1 PE=1 SV=2	0	174.72	42	18	86	18	573	61	5.87	0.649867773	0.001492588	190052221.4	292447524.4	202098104.4	181067728.8	186990830.9	310844454.1	270916722.4	295581396.8
High	P54886	ALDH18A1	Delta-1-pyrroline-5-carboxylate synthase OS=Homo sapiens OX=9606 GN=ALDH18A1 PE=1 SV=2	0	173.825	28	17	69	17	795	87.2	7.12	0.614300965	0.005027833	41683962.44	67855928.63	48889713.44	35672418.66	40489755.22	72444609.41	63270472.84	67852703.63
High	P08195	SLC3A2	4F2 cell-surface antigen heavy chain OS=Homo sapiens OX=9606 GN=SLC3A2 PE=1 SV=3	0	171.063	32	15	78	15	630	68	5.01	0.720979323	0.011056666	92795680.57	128707825	105107099.4	89013594.7	84266347.56	132598731.4	134686218.2	118838525.5
High	P21291	CSRP1	Cysteine and glycine-rich protein 1 OS=Homo sapiens OX=9606 GN=CSRP1 PE=1 SV=3	0	167.016	53	10	68	10	193	20.6	8.57	0.656579836	0.003353463	138723654.3	211282233.7	160156636.1	129916459.2	126097867.7	202729908.2	214110974.9	217005818
High	P63241	EIF5A	Eukaryotic translation initiation factor 5A-1 OS=Homo sapiens OX=9606 GN=EIF5A PE=1 SV=2	0	166.72	68	10	81	10	154	16.8	5.24	0.567917963	0.001372182	171642853.5	302231774	189619876.3	155742152.7	169566531.7	326757226.5	281407084.2	298531011.2
High	Q96HE7	ERO1A	ERO1-like protein alpha OS=Homo sapiens OX=9606 GN=ERO1A PE=1 SV=2	0	165.932	31	12	54	12	468	54.4	5.68	0.67858232	0.005854527	34095401.48	50245048.35	38438646.56	31930884.53	31916673.34	54414251.75	47893783.5	48427109.81
High	P06737	PYGL	Glycogen phosphorylase, liver form OS=Homo sapiens OX=9606 GN=PYGL PE=1 SV=4	0	165.562	31	23	78	18	847	97.1	7.17	0.735200678	0.020618041	80629927.22	109670637.8	88248019.81	87770008.87	65871752.97	111236835.4	104601444.6	113173633.4
High	P46459	NSF	Vesicle-fusing ATPase OS=Homo sapiens OX=9606 GN=NSF PE=1 SV=3	0	165.456	31	20	67	20	744	82.5	6.95	0.695093098	0.005891669	37707496.84	54248124.41	41693042.25	33358935.83	38070512.44	58071011.69	51818721.25	52854640.28
High	Q02790	FKBP4	Peptidyl-prolyl cis-trans isomerase FKBP4 OS=Homo sapiens OX=9606 GN=FKBP4 PE=1 SV=3	0	163.241	46	14	63	14	459	51.8	5.43	0.64748252	0.039272415	35062149.89	54151500.35	45517153.44	28636673.67	31032622.56	60919920.88	52288886.13	49245694.06
High	Q9P0K7	RAI14	Ankycorbin OS=Homo sapiens OX=9606 GN=RAI14 PE=1 SV=2	0	161.307	22	20	75	20	980	110	6.21	0.824306654	0.044852177	74795872.28	90737921.33	83765320.75	68790990.78	71831305.31	96922313.69	87044059.81	88247390.5
High	Q9UQ80	PA2G4	Proliferation-associated protein 2G4 OS=Homo sapiens OX=9606 GN=PA2G4 PE=1 SV=3	0	160.124	42	14	64	14	394	43.8	6.55	0.650292433	0.006882591	83179484.4	127910890.9	90216896.11	79884197.09	79437360	129086318.3	141132175.9	113514178.5
High	Q96CW1	AP2M1	AP-2 complex subunit mu OS=Homo sapiens OX=9606 GN=AP2M1 PE=1 SV=2	0	159.14	49	17	68	17	435	49.6	9.54	0.732016459	0.038325196	95514885.15	130481881.8	104423585.8	82504664.41	99616405.27	148148258.2	116210709.4	127086677.8
High	P62191	PSMC1	26S proteasome regulatory subunit 4 OS=Homo sapiens OX=9606 GN=PSMC1 PE=1 SV=1	0	158.008	46	15	62	14	440	49.2	6.21	0.791142839	0.042975623	54265509.34	68591291.83	59395768.19	50785449.72	52615310.13	76810951.63	63672993.16	65289930.72
High	P25786	PSMA1	Proteasome subunit alpha type-1 OS=Homo sapiens OX=9606 GN=PSMA1 PE=1 SV=1	0	157.937	50	12	64	12	263	29.5	6.61	0.814238753	0.032149519	148613642.2	182518507.9	167947534.7	139476761.2	138416630.8	186210828.1	187065349.1	174279346.5
High	O00116	AGPS	Alkyldihydroxyacetonephosphate synthase, peroxisomal OS=Homo sapiens OX=9606 GN=AGPS PE=1 SV=1	0	157.11	35	15	42	15	658	72.9	7.34	0.710118779	0.027672038	29531331.48	41586467.42	35209228.13	25466318.25	27918448.06	45378174.59	40954507.84	38426719.81
High	Q9UM54	MYO6	Unconventional myosin-VI OS=Homo sapiens OX=9606 GN=MYO6 PE=1 SV=4	0	156.856	18	18	56	18	1294	149.6	8.53	0.65417307	0.001987771	26089971.01	39882367.85	24251904.97	29847279.31	24170728.75	39882250	39200255.44	40564598.13
High	Q8N8S7	ENAH	Protein enabled homolog OS=Homo sapiens OX=9606 GN=ENAH PE=1 SV=2	0	156.516	30	15	60	15	591	66.5	6.93	0.788821476	0.044017605	78459058.92	99463644.6	86512746.41	78545933.44	70318496.91	110002278.6	91318741.44	97069913.75
High	P09651	HNRNPA1	Heterogeneous nuclear ribonucleoprotein A1 OS=Homo sapiens OX=9606 GN=HNRNPA1 PE=1 SV=5	0	154.016	30	11	70	9	372	38.7	9.13	0.622856579	0.007852754	130245867.3	209110526.7	152334431.9	108097455.9	130305714.1	228297477.1	200637352.9	198396750.1
High	P61978	HNRNPK	Heterogeneous nuclear ribonucleoprotein K OS=Homo sapiens OX=9606 GN=HNRNPK PE=1 SV=1	0	153.767	42	16	96	16	463	50.9	5.54	0.760622236	0.04415228	221048072.8	290614791.7	249517254.3	204541414.7	209085549.4	327125213.8	261660023	283059138.4
High	O14818	PSMA7	Proteasome subunit alpha type-7 OS=Homo sapiens OX=9606 GN=PSMA7 PE=1 SV=1	0	150.674	59	13	62	13	248	27.9	8.46	0.758249292	0.02733001	122462049.7	161506315.9	142352048.7	110663180.8	114370919.6	163785794.3	150771545.2	169961608.3
High	P24821	TNC	Tenascin OS=Homo sapiens OX=9606 GN=TNC PE=1 SV=3	0	150.275	12	18	43	18	2201	240.7	4.89	0.700110747	0.007876996	21617133.45	30876734.21	25139513.63	20462411.88	19249474.84	31805660.13	29896626.63	30927915.88
High	O75436	VPS26A	Vacuolar protein sorting-associated protein 26A OS=Homo sapiens OX=9606 GN=VPS26A PE=1 SV=2	0	150.174	46	11	59	10	327	38.1	6.57	0.775059915	0.025972067	60432163.79	77970957.6	67352294.13	61926667.34	52017529.89	82716146.88	75440010.06	75756715.88
High	P61163	ACTR1A	Alpha-centractin OS=Homo sapiens OX=9606 GN=ACTR1A PE=1 SV=1	0	146.506	42	12	43	6	376	42.6	6.64	0.776078429	0.017959876	31108492.05	40084211.73	34644654	29032519.84	29648302.31	42771371.44	39849904.63	37631359.13
High	P52789	HK2	Hexokinase-2 OS=Homo sapiens OX=9606 GN=HK2 PE=1 SV=2	0	142.299	25	19	69	15	917	102.3	6.05	0.708327059	0.042437058	21310274.53	30085359.95	23798664.31	17811928.13	22320231.16	34181670.06	25930959.72	30143450.06
High	Q02878	RPL6	60S ribosomal protein L6 OS=Homo sapiens OX=9606 GN=RPL6 PE=1 SV=3	0	141.795	43	14	71	14	288	32.7	10.58	0.799203052	0.019432853	322035905.3	402946290.5	353325635.9	301187262.7	311594817.3	431382576.4	391286489.7	386169805.5
High	O95479	H6PD	GDH/6PGL endoplasmic bifunctional protein OS=Homo sapiens OX=9606 GN=H6PD PE=1 SV=2	0	139.873	20	12	38	12	791	88.8	7.3	0.766500101	0.039232864	31152100.19	40642004.02	35239393.75	32143213.63	26073693.19	42034645.25	37403089.88	42488276.94
High	P49189	ALDH9A1	4-trimethylaminobutyraldehyde dehydrogenase OS=Homo sapiens OX=9606 GN=ALDH9A1 PE=1 SV=3	0	139.187	36	16	76	16	494	53.8	5.87	0.798085645	0.033368964	106960080	134020804.2	119004872.7	103067790.7	98807576.5	145351527.6	125728558.9	130982326.2
High	P23284	PPIB	Peptidyl-prolyl cis-trans isomerase B OS=Homo sapiens OX=9606 GN=PPIB PE=1 SV=2	0	138.992	56	13	113	13	216	23.7	9.41	0.694231995	0.041185039	653251979.7	940970719.4	801214016.8	575552438.7	582989483.6	1036028639	822994396.9	963889122.5
High	P27695	APEX1	DNA-(apurinic or apyrimidinic site) endonuclease OS=Homo sapiens OX=9606 GN=APEX1 PE=1 SV=2	0	137.599	48	10	40	10	318	35.5	8.12	0.683850637	0.013425058	32069496.24	46895468.83	35067324.13	33314715.84	27826448.75	52074286.06	45905461.94	42706658.5
High	Q9BZQ8	NIBAN1	Protein Niban 1 OS=Homo sapiens OX=9606 GN=NIBAN1 PE=1 SV=1	0	134.677	17	13	45	13	928	103.1	4.78	0.721086038	0.005336756	50766836.47	70403299.78	50827569.88	56147651.97	45325287.58	68226218.44	69160256.38	73823424.52
High	P38117	ETFB	Electron transfer flavoprotein subunit beta OS=Homo sapiens OX=9606 GN=ETFB PE=1 SV=3	0	134.421	51	14	67	14	255	27.8	8.1	0.695687817	0.002509734	96816886.24	139167143.4	108649237.9	88969614.43	92831806.41	142141683.8	139317691.8	136042054.6
High	P17812	CTPS1	CTP synthase 1 OS=Homo sapiens OX=9606 GN=CTPS1 PE=1 SV=2	0	132.612	31	16	52	16	591	66.6	6.46	0.795697899	0.013487738	65218568.38	81963982.13	65083503.75	62160608.64	68411592.75	86833774.44	83966440.31	75091731.63
High	P56199	ITGA1	Integrin alpha-1 OS=Homo sapiens OX=9606 GN=ITGA1 PE=1 SV=2	0	132.205	17	17	50	17	1179	130.8	6.29	0.80581462	0.021004029	59982900.78	74437593.08	64912381.88	58123672.87	56912647.58	79223605.81	68843967.38	75245206.06
High	P62820	RAB1A	Ras-related protein Rab-1A OS=Homo sapiens OX=9606 GN=RAB1A PE=1 SV=3	0	128.797	64	12	55	5	205	22.7	6.21	2.166620397	0.006317042	108864580.7	50246264.11	124439401.4	97815658.33	104338682.4	65685465.41	40706929.69	44346397.25
High	P30085	CMPK1	UMP-CMP kinase OS=Homo sapiens OX=9606 GN=CMPK1 PE=1 SV=3	0	128.53	61	10	59	10	196	22.2	5.57	0.793337483	0.021900269	57575278.21	72573500.46	64945923.13	55705269.56	52074641.94	75022415.88	69831813.38	72866272.13
High	Q8TAT6	NPLOC4	Nuclear protein localization protein 4 homolog OS=Homo sapiens OX=9606 GN=NPLOC4 PE=1 SV=3	0	127.943	26	12	38	12	608	68.1	6.38	0.67786849	0.044949378	19281787.98	28444732.67	19269332.39	18160412.55	20415619	34457782.75	24042338.63	26834076.63
High	Q9NSD9	FARSB	Phenylalanine--tRNA ligase beta subunit OS=Homo sapiens OX=9606 GN=FARSB PE=1 SV=3	0	127.903	31	16	65	16	589	66.1	6.84	0.717907895	0.017134663	61022615.51	85000619.04	69266732.31	54765085.03	59036029.19	93500036	82195602.5	79306218.63
High	P00568	AK1	Adenylate kinase isoenzyme 1 OS=Homo sapiens OX=9606 GN=AK1 PE=1 SV=3	0	126.064	51	8	41	8	194	21.6	8.63	0.480446924	9.62111E-05	38406332.43	79938762.21	42116730.5	39674413.38	33427853.41	79892944	79002310.5	80921032.13
High	P05387	RPLP2	60S acidic ribosomal protein P2 OS=Homo sapiens OX=9606 GN=RPLP2 PE=1 SV=1	0	125.616	77	7	52	7	115	11.7	4.54	0.622474304	0.02243449	121831737.1	195721713.1	152611364.5	84673978.63	128209868.3	204172439	187669375.3	195323325.1
High	P15880	RPS2	40S ribosomal protein S2 OS=Homo sapiens OX=9606 GN=RPS2 PE=1 SV=2	0	125.496	50	13	63	13	293	31.3	10.24	1.352431337	0.035475696	355071734	262543261.4	394031380.8	317871702.1	353312118.9	301003605.6	234697367.6	251928811.1
High	P04899	GNAI2	Guanine nucleotide-binding protein G(i) subunit alpha-2 OS=Homo sapiens OX=9606 GN=GNAI2 PE=1 SV=3	0	124.677	34	9	48	6	355	40.4	5.54	0.580079696	0.023507074	14789086.3	25494921.46	19089842.44	10103035.52	15174380.94	28453840.5	23562230.56	24468693.31
High	O43175	PHGDH	D-3-phosphoglycerate dehydrogenase OS=Homo sapiens OX=9606 GN=PHGDH PE=1 SV=4	0	124.176	30	13	52	13	533	56.6	6.71	0.618592924	0.010409356	35317180.25	57092764.65	44330508.75	32896845.93	28724186.06	59220896	55986060.63	56071337.31
High	O15144	ARPC2	Actin-related protein 2/3 complex subunit 2 OS=Homo sapiens OX=9606 GN=ARPC2 PE=1 SV=1	0	123.759	53	13	72	13	300	34.3	7.36	0.764355789	0.039175068	264140642.5	345572894.5	302637062.7	234546528.6	255238336.1	358001810.1	310250175.2	368466698.1
High	P61221	ABCE1	ATP-binding cassette sub-family E member 1 OS=Homo sapiens OX=9606 GN=ABCE1 PE=1 SV=1	0	122.272	27	12	52	12	599	67.3	8.34	0.765068907	0.008023521	46787627.13	61154788.42	50366184.25	48031308.25	41965388.88	64070885	58939933.38	60453546.88
High	Q12768	WASHC5	WASH complex subunit 5 OS=Homo sapiens OX=9606 GN=WASHC5 PE=1 SV=1	0	120.503	14	11	37	11	1159	134.2	6.98	0.664920889	0.011754946	6885270.438	10355022.01	6400272.406	6371172.688	7884366.219	11534056.97	10048457.56	9482551.5
High	P48739	PITPNB	Phosphatidylinositol transfer protein beta isoform OS=Homo sapiens OX=9606 GN=PITPNB PE=1 SV=2	0	120.265	59	14	63	13	271	31.5	6.87	0.778899585	0.045083988	51055792.53	65548619.48	54960083.91	52183740.19	46023553.5	73254360.84	58440107.41	64951390.19
High	P42765	ACAA2	3-ketoacyl-CoA thiolase, mitochondrial OS=Homo sapiens OX=9606 GN=ACAA2 PE=1 SV=2	0	120.017	44	12	46	12	397	41.9	8.09	0.680777418	0.022694954	49027543.43	72016994.3	59092590.19	38837414.23	49152625.88	76698615.56	67912267.22	71440100.13
High	Q9BT78	COPS4	COP9 signalosome complex subunit 4 OS=Homo sapiens OX=9606 GN=COPS4 PE=1 SV=1	0	118.907	38	12	33	12	406	46.2	5.83	0.667177267	0.022555377	25769718.9	38624995.46	28729702.94	20788678.52	27790775.25	43292534.94	34596129.31	37986322.13
High	P09493	TPM1	Tropomyosin alpha-1 chain OS=Homo sapiens OX=9606 GN=TPM1 PE=1 SV=2	0	118.646	37	14	71	4	284	32.7	4.74	0.671234883	0.036759026	41348209.21	61600209.17	49411144	34792321.13	39841162.5	58283716.5	55139356	71377555
High	P17931	LGALS3	Galectin-3 OS=Homo sapiens OX=9606 GN=LGALS3 PE=1 SV=5	0	117.974	34	8	83	8	250	26.1	8.56	0.816015992	0.03834766	249714548.6	306016733.8	274001387.4	251492163.4	223650094.9	326286930.9	286733096.4	305030174.1
High	Q99623	PHB2	Prohibitin-2 OS=Homo sapiens OX=9606 GN=PHB2 PE=1 SV=2	0	117.313	54	14	59	14	299	33.3	9.83	0.765143609	0.036009371	161355488.5	210882619.5	181700975.2	148518905.8	153846584.6	229248671.6	187834853.6	215564333.2
High	Q9UHD8	SEPTIN9	Septin-9 OS=Homo sapiens OX=9606 GN=SEPTIN9 PE=1 SV=2	0	116.729	33	14	58	14	586	65.4	8.97	0.770728621	0.02482277	94471434.98	122574188.2	110292876.3	87571137.56	85550291.06	121686652.4	124941917.4	121093994.8
High	P50993	ATP1A2	Sodium/potassium-transporting ATPase subunit alpha-2 OS=Homo sapiens OX=9606 GN=ATP1A2 PE=1 SV=1	0	116.531	15	11	50	1	1020	112.2	5.66	0.177855082	0.018839334	97027.48014	545542.3542	2574.313965	2574.313965	285933.8125	674984.8125	526736.875	434905.375
High	P49591	SARS1	Serine--tRNA ligase, cytoplasmic OS=Homo sapiens OX=9606 GN=SARS1 PE=1 SV=3	0	116.452	35	13	39	13	514	58.7	6.43	0.625956333	0.002460854	38314582.95	61209673.81	43198142.88	39668758.66	32076847.31	62489749.63	61353800.25	59785471.56
High	P36551	CPOX	Oxygen-dependent coproporphyrinogen-III oxidase, mitochondrial OS=Homo sapiens OX=9606 GN=CPOX PE=1 SV=3	0	115.974	32	11	31	11	454	50.1	8.25	0.686204416	0.0488878	14236228.75	20746338.01	18227249.25	11297045.44	13184391.56	22784628.63	19195688.63	20258696.78
High	Q92973	TNPO1	Transportin-1 OS=Homo sapiens OX=9606 GN=TNPO1 PE=1 SV=2	0	115.495	20	15	53	12	898	102.3	4.98	0.746954694	0.026043297	22878124.3	30628530.07	24784451.19	25248893.78	18601027.92	29941122.03	31976028.63	29968439.56
High	P09429	HMGB1	High mobility group protein B1 OS=Homo sapiens OX=9606 GN=HMGB1 PE=1 SV=3	0	115.108	26	8	39	7	215	24.9	5.74	0.820069436	0.01121481	91579711.44	111673118.6	92992175.5	90278858.38	91468100.44	117697856.8	103004627.5	114316871.6

Note: Molecular targets are sorted by sum PEP score and FDR confidence, with the first 100 identities listed at top candidates.

Table S3. A list of primary antibodies and applied concentrations.

Antibody name	*Cat. log number*	*Concentration used*
Rabbit monoclonal anti-phospho-ATM (S1981)	Abways cat. no. CY5111	1:1000 (WB)
Rabbit monoclonal anti-ATM	Abways cat. no. CY5207	1:1000 (WB)
Rabbit monoclonal anti-phospho-Histone H2AX (Ser139)	Cell Signaling cat. no. 9718	1:1000 (WB)
Rabbit monoclonal anti-Histone H2AX	Abcam cat. no. ab229914	1:1000 (WB)
Rabbit polyclonal anti-phospho-p38	ABclonal cat. no. AP0526	1:1000 (WB)
Rabbit polyclonal anti-p38	Cell Signaling cat. no. 9212	1:1000 (WB)
Rabbit monoclonal anti-phospho-Akt (Ser473) (D9E)	Cell Signaling cat. no. 4060	1:1000 (WB)
Rabbit monoclonal anti-AKT1 + AKT2	Abcam cat. no. ab188099	1:1000 (WB)
Rabbit monoclonal anti-phospho-mTOR (Ser2448) (D9C2)	Cell Signaling cat. no. 5536	1:1000 (WB)
Rabbit monoclonal anti-mTOR (7C10)	Cell Signaling cat. no. 2983	1:1000 (WB)

Rabbit monoclonal anti-phospho-p53-S9	ABclonal cat. no. APO985	1:1000 (WB)
Rabbit monoclonal anti-p53	ABclonal cat. no. A25915	1:1000 (WB)
Rabbit monoclonal anti-p21 antibody	Abcam cat. no. Ab109520	1:1000 (WB)
Mouse monoclonal anti-IL8	Abways cat. no. AB3122	1:1000 (WB)
Rabbit monoclonal anti-MMP3	Proteintech cat. no. 66338-1-IG-	1:1000 (WB)
Rabbit monoclonal anti-HSP70	Proteintech cat. no. HY-P80708	1:1000 (WB)
Rabbit polyclonal anti-IL6	Abmart cat. no. TD6087S	1:1000 (WB)
Mouse anti-human p16(G175-1239)	BD Pharmingen cat. no. 554079	1:1000 (WB)
Rabbit monoclonal anti-GAPDH	Abways cat. no. AB0037	1:1000 (WB)
Mouse monoclonal anti-β-actin	Proteintech cat. no. 66009-1-Ig	1:1000 (WB)
Rabbit polyclonal anti-F4/80	Proteintech cat No. 29414-1-AP	1:800 (IHC)
Rabbit polyclonal anti-NF-kB p65	ABclonal cat. no. (A19653)	1:5000 (WB) 1:500 (IHC)
Rabbit monoclonal anti-NRF2	ABclonal cat. no. (A3577)	1:1000 (WB)

Rabbit monoclonal anti-COL3A1	Cell Signaling cat. no.73034	1:1000 (WB)
Rabbit monoclonal anti-COL1A1 (E8F4L) XP®	Cell Signaling cat. no.72026	1:1000 (WB)
Rabbit polyclonal anti-Lamin A/C	Proteintech cat No. 10298-1-AP	1:1000 (WB)
Alexa Fluor® 700 anti-mouse CD45 antibody	Biolegend Cat. No. 103127	1:100 ((Flow)
Brilliant Violet 510™ anti-mouse/human CD11b antibody	Biolegend Cat. No. 101245	1:100 (Flow)
PE anti-mouse F4/80 antibody	Biolegend Cat. No. 123109	1:100 (Flow)
Brilliant Violet 421™ anti-mouse Ly-6G/Ly-6C (Gr-1) antibody	Biolegend Cat. No. 108433	1:100 (Flow)
FITC anti-mouse I-A/I-E (MHC-II) antibody	Biolegend Cat. No. 107605	1:100 (Flow)

APC anti-mouse CD206 (MMR) Antibody	Biolegend Cat. No. 141707	1:100(Flow)

Table S4. A list of quantitative RT-PCR primers.

Gene	*Forward primer (5'-3')*	*Reverse primer (5'-3')*
IL6	TACCCCCAGGAGAAGATTCC	TTTTCTGCCAGTGCCTCTTT
IL8	GTGCAGTTTTGCCAAGGAGT	CTCTGCACCCAGTTTTCCTT
IL1α	TTGTGATCTTGGGTTGGTCA	ATGTTGGTGGGTGACTGGAT
IL1β	GGGCCTCAAGGAAAAGAATC	TTCTGCTTGAGAGGTGCTGA
GM-CSF	CCCCAGTCACCTGCTGTTAT	TGGAATCCTGAACCCACTTC
CXCL1	AGGGAATTCACCCCAAGAAC	TGGATTTGTCACTGTTCAGCA
CXCL3	GCAGGGAATTCACCTCAAGA	GGTGCTCCCCTTGTTCAGTA
RPL13A	GTACGCTGTGAAGGCATCAA	CGCTTTTTCTTGTCGTAGGG
mIL6	CCAGTTGCCTTCTTGGGACT	GTCTCCTCTCCGGACTTGTG
mIL1α	AACGTCAAGCAACGGGAAGA	TGCTGATCTGGGTTGGATGG
mIL1β	AGAGCCCATCCTCTGTGACT	GCTTGGGATCCACACTCTCC
mCXCL1	CAGAGCTTGAAGGTGTTGCC	CAAGGGAGCTTCAGGGTCAA
mCXCL2	TCCAGAGCTTGAGTGTGACG	GCAAACTTTTTGACCGCCCT
mCXCL7	GCCTGCCCACTTCATAACCT	ATCTGCAGCGCAGTTCGATA
mGAPDH	GAGAGTGTTTCCTCGTCCCG	ACTGTGCCGTTGAATTTGCC
